# Do indigenous people get left behind? An innovative methodology for measuring the unmeasurable economic conditions and poverty from the poorest region of Luzon, Philippines

**DOI:** 10.1016/j.heliyon.2024.e41076

**Published:** 2024-12-24

**Authors:** Emmanuel A. Onsay, Jomar F. Rabajante

**Affiliations:** a Graduate School, University of the Philippines Los Baños, Laguna, 4030, Philippines; b Partido Institute of Economics, Partido State University, Camarines Sur, 4422, Philippines

**Keywords:** Indigenous communities, Multidimensional poverty, Socioeconomic modelling, Data analytics, Advanced econometrics, Policy, Philippines

## Abstract

One of the most vulnerable, neglected, and marginalized groups in society is indigenous communities. Their socioeconomic circumstances are intricate and varied. The oldest societal issue ever is poverty, which is also the hardest to solve. It is multifaceted and immeasurable, especially for the Agta Tabangnon, our Indigenous people. Research on indigenous peoples is qualitative in nature, whereas research on poverty is typically general, subject to significant sampling errors, and meant to inform national policy. Therefore, it is essential for economic development to measure multidimensional poverty and simulate the socioeconomic conditions for each tribe through full enumeration and disaggregation. This work puts forth fresh approaches to quantify the incalculable multifaceted poverty and socioeconomic conditions: (i) a thorough statistical analysis using diagnostic and descriptive analytics to examine socioeconomic situations; (ii) combining sophisticated econometrics and predictive analytics to measure multidimensional poverty; and (iii) integrating machine learning to model socioeconomic situations and prescriptive analytics to develop policy. Analysis reveals that poverty among tribes is closely tied to income, livelihood, and education, impacting various aspects of daily life, including housing, water, sanitation, health, and nutrition. The study develops multidimensional poverty indicators crucial for promoting economic development and poverty reduction among Indigenous communities. The study underscores the urgency of targeted policies and programs to address multidimensional deprivations faced by Indigenous communities, emphasizing the importance of data-driven approaches in improving welfare and promoting equitable socio-economic progress. Other researchers worldwide could replicate, reproduce, or reuse the suggested methods to assist indigenous peoples in reducing poverty and enhancing their well-being. It can be freely applied to focus policy on tribal economy, which has multiple dimensions and necessitates various forms of development initiatives.

## Graphical abstract

**Figure:**
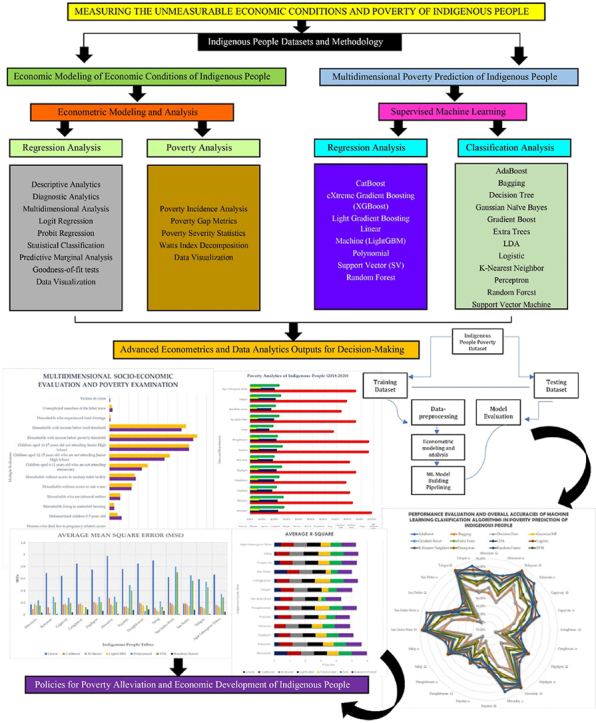

**Table:** Specifications table

Subject area	Economics and Finance
**More specific subject area**	Poverty Economics, Applied Econometrics, Data Analytics, Development Studies
**Name of your method**	1. Statistical Analysis (Diagnostic and Descriptive analytics) 2. Advanced Econometrics and Predictive Analytics (Logistic and Probit Regression) 3. Machine Learning Regression (CatBoost, XGBoost, LightGBM, Polynomial, Support Vector, Random Forest) 4. Machine Learning Classification (AdaBoost, Bagging, Decision Tree Gaussian Naïve Bayes, Gradient Boost, Extra Trees, LDA, Logistic, K-Nearest Neighbor, Perceptron, Random Forest, and Support Vector Machine) 5. Prescriptive Analytics (Policy Mapping and Policy Targeting)
**Name and reference of original method**	1. Haughton, Jonathan, and Shahidur R. Khandker. Handbook on poverty + inequality. World Bank Publications, 2009. 2. Onsay, EA., Rabajante J. Measuring the Unmeasurable Multidimensional Poverty for Economic Development: Datasets, Algorithms, and Models from the Poorest Region of Luzon, Philippines. Data in Brief Journal. https://doi.org/10.1016/j.dib.2024.110150 (2024) 3. Reyes, Celia M., and Lani E. Valencia. "CBMS as a Tool for Decentralised Poverty Monitoring." Poverty Monitoring in Asia (2008): 301. 4. Sobreviñas, Alellie Borel. "The community-based monitoring system (CBMS): An investigation of its usefulness in understanding the relationship between international migration and poverty in the Philippines." PhD diss., University of Antwerp, 2017. 5. Muñetón-Santa, Guberney, and Luis Carlos Manrique-Ruiz. "Predicting multidimensional poverty with machine learning algorithms: an open data source approach using spatial data." Social Sciences 12, no. 5 (2023): 296. 6. Hastie, Trevor, Robert Tibshirani, Jerome H. Friedman, and Jerome H. Friedman. The elements of statistical learning: data mining, inference, and prediction. Vol. 2. New York: springer, 2009. 7. Mujumdar, Aishwarya, and V. Vaidehi. "Diabetes prediction using machine learning algorithms." Procedia Computer Science 165 (2019): 292–299.
**Resource availability**	Dataset: Measuring the Unmeasurable Multidimensional Socio-Economic Deprivations and Poverty Predictions: Indigenous People Datasets for Econometrics, Machine Learning, and Quantitative Social Science Modeling DOI: https://doi.org/10.7910/DVN/QSZKUP Repository: Harvard Dataverse

## 1 Method details

### 1.1 Introduction to methods

As old as poverty is the indigenous people, thus data and methods must be made available for public consumption to alleviate poverty and promote economic development [1]. They have been a part of civilization for generations and are very important. In the Philippines, they have rights to ancestral territories and lands, social justice, human rights, and self-governance and empowerment in addition to cultural integrity. It follows that official protection and public respect for Native communities are imperative [2]. In order to alleviate their poverty and promote growth, it is necessary to preserve their culture and habits [3]. One of the most persistent social problems that has never been fully resolved is poverty. According to Haughton and Khandker (2009), it hinders societal advancement and economic success. It is challenging to control and undo [4]. Various economists and data scientists have attempted to elucidate the reasons behind poverty and the necessary adjustments. Nevertheless, measuring poverty in remote places or among indigenous populations is more challenging, and there is a paucity of published research on the topic.

The Philippines is home to diverse indigenous communities. As of right now, no comprehensive studies have been conducted on the quantitative evaluation of multidimensional poverty among the indigenous population in the Philippines. The way in which the prevalence of poverty, the advancement of the economy of our indigenous people, and their social development, particularly in the Bicol region is insufficient. Furthermore, very few studies have used complete enumeration methodologies to investigate the extent of poverty experienced by our indigenous people. However, an evaluation carried out in developed countries such as Australia revealed that significant barriers exist for Indigenous people in selecting commercial and economic development options that will promote their social and economic advancement within society [5]. In order to support logical decision-making in modern society, approaches that address indigenous people communities from social science perspectives are essential for opportunity analysis and problem assessment [6].

There are several different indigenous communities living in the Philippines. It is imperative to emphasize that the subject matter covered in this research study is the Agta Tabangnon community who live in Mt. Isarog, Goa, Camarines Sur. The data pertaining to indigenous people in this study was obtained using a community-based monitoring system and covers the years 2018–2020. The Agta Tabangnon are indigenous to this region of the Bicol Region in the Philippines, and they live on the slopes of Mount Isarog. The Agta-Tabangnon were the first people to live in the forest-edge communities of Mount Isarog. The Municipality of Goa had twelve Agta Communities as of 2019 [1,3]. Agta Tabangnon people are known for their low stature, dark skin, kinky hair, big noses, thick lips, and deep-set eyes. Nonetheless, these traits have changed as a result of marriages with outsiders and lowlanders [52]. Lightweight building materials including nipa, sawali, coconut leaves, and abaca are used to build their homes. Four strong poles, rafters made of secondary growth trees, and a thatched roof are the conventional components of a hut. There is a section of the house that is three feet above the floor, with a floor made of bamboo slats that doubles as a platform for eating and sleeping. The typical indigenous people dwelling is modest and ephemeral, reflecting their outlook on life. Materials used in their traditional garments include balete tree bark, pineapple fibers, and balakbak. Indigenous people women started wearing longer tapis and concealing their breasts after accepting Christianity, but both bahag and tapis were originally worn by men and women [3,51,[53], [54], [55]].

Twenty-five indigenous people families in the Northern Philippines were examined and found that they have different socioeconomic features and less access to technology than people living in rural and urban areas [56]. The main source of income for Aeta people is farming, while some families gain from social handouts that the government has instituted. Since before colonization, indigenous peoples have coexisted peacefully in the area. The Agta Tabangnon are characterized by their low stature, dark skin, kinky hair, deep-set eyes, big nose, and thick lips. However, as a result of marriages with foreigners and lowlanders, these traits have changed [52]. When it comes to poverty, the number of indigenous people in the region living below the poverty line is higher than that of non-IP populations. While poverty is common in the area, it is clear that IPs experience poverty at a higher rate than non-IPs. In terms of multifaceted socioeconomic deprivations, IPs seemed to be less fortunate than non-IPs [1,3].

In the sphere of natural sciences, several researchers have attempted to quantify the unquantifiable [[7], [8], [9], [10]]. However, social issues and economic issues are challenging to quantify, assess, and sometimes unquantifiable [11,15]. In light of the aforementioned, we are putting forth innovative techniques and methods to measure the unmeasurable, multifaceted, and varied socioeconomic circumstances of our indigenous population. These techniques will be used as a basis for developing policies aimed at reducing poverty and promoting economic growth.

Disaggregated data that can be utilized for planning is gathered, examined, and validated using a technology-based system known as the Community-Based Monitoring System. Communities are given the chance to be involved in the process when program implementation and impact monitoring are being carried out locally [58]. The CBMS databases have enough data to measure the multifaceted dimensions of development, inequality, and poverty. Since the Community-Based Monitoring System (CBMS) is being deployed countrywide, the statistics we present here are comparable to other datasets in the Philippines [14]. Additionally, the CBMS network is operational in over 20 countries globally, facilitating the creation of comparable datasets for use by other investigators [57]. These datasets can be used in conjunction with other datasets to evaluate progress towards the Sustainable Development Goals (SDGs), which are important to achieve by 2030 [13]. However, this research's approaches for evaluating poverty do not necessitate data gathering in the same way as CBMS. Immediate replication may be possible if the variables indicated in this paper are present in other nations or locations. Communities of indigenous people in the Philippines see little value in the surveys that are currently available. Indigenous groups are frequently underrepresented and lack social, demographic, and economic statistics. The Philippine Statistics Authority does not expressly tailor its existing surveys to serve indigenous groups. For the purpose of promoting economic development and reducing poverty, policymakers will find this approach article to be highly significant. This methodological article was conducted in the Philippines by utilizing a complete enumeration of the entire indigenous population from the 12 tribes of Mt. Isarog.

Moreover, indigenous groups have unique knowledge, a rich cultural legacy, and environmentally friendly customs that can help the entire community. Not only is it morally right to preserve their culture and way of life, but it also offers a chance for mutual enrichment and learning. We may create specialized policies and actions that respect indigenous communities' traditional values and advance inclusive development by having a thorough understanding of their socioeconomic conditions. Furthermore, indigenous peoples have a strong bond with nature and frequently live in ecologically significant places. Their sustainable practices and traditional ecological knowledge can support biodiversity preservation and environmental conservation. We can ensure a sustainable use of resources for future generations by fostering a harmonious interaction between human communities and the environment through tackling poverty and supporting economic development among indigenous cultures [1,2].

Predictive analytics and advanced econometrics have not been used in any research to measure poverty in Mt. Isarog where our indigenous tribes (Agta Tabangnon) are situated. With a poverty incidence of 38.7 %, the Bicol area is the poorest province in the Bicol region, which is the poorest region in Luzon [12]. Because of this, the dataset is essential for combating multifaceted poverty and fostering the economic development of our indigenous people—not only in this area but also globally—by applying the models and indicators employed in this research. The core focus of our paper lies in the methodology introduced to ensure reproducibility. While we present research findings succinctly, they are a condensed representation of the results. A significant part of the article is dedicated to elucidating the computational processes of various methods, with a detailed analysis of the outcomes available in a separate publication linked on each finding. Our objective is to emphasize and spotlight these computational approaches. The results we showcase, including insights into economic conditions and the multidimensional poverty status of the study's residents, serve as practical examples of deploying these methods effectively. Our primary goal is to delineate the pros and cons of diverse calculation methods with the aim of enabling researchers in the Philippines and globally to utilize them for enhancing the well-being of their indigenous communities.

Furthermore, economic growth and the alleviation of poverty are important goals on a local, national, and international scale. We can aid in the accomplishment of the Sustainable Development Goals of the UN, especially Goal 1 (No Poverty) and Goal 8 (Decent Work and Economic Growth), by concentrating on poverty among indigenous communities. Sustainable economic advancement and more efficient methods of reducing poverty can result from acknowledging the unique difficulties faced by indigenous populations and adjusting initiatives accordingly. These initiatives could eradicate poverty, reduce hunger, advance high-quality education, promote excellent health, and enhance community well-being [13].

## 2 Materials and methods

### 2.1 Source of data

The Community-Based Monitoring System (CBMS) in Goa, Camarines Sur, developed the dataset. This is a census conducted by the local government once every three years in accordance with Republic Act 11315 (The CBMS Law). The local government entity is the owner of the data. They conduct the census for the entire population within their jurisdiction and are responsible for data enumeration and validation in partnership with the Philippine Statistics Authority and academia. Data processes include the rigorous training of data enumerators, timely data gathering, technology-based data collection, and periodic evaluation of raw data. Data validation is performed to ensure accuracy and involves several key processes. We perform data cleaning to rectify errors, double-entry verification for accuracy, range and consistency checks, the application of validation rules, cross-field validation, duplicate detection, geospatial validation, and metadata validation. Assessing data quality and documenting outcomes are crucial for transparency. We carry out all these steps; thus, they enhance the reliability of the collected data. The overall purpose of this system is to provide policymakers with the necessary data to track the regional economy. It can be used as a guide to develop successful programs and policies that promote community development and lessen poverty, as well as to pinpoint the underlying causes of poverty. The household dataset and the member dataset are the two (2) available datasets. The data validation process was finished in 2020, and the survey started in 2018. The cross-sectional data set included information from 2018 to 2020. Data collection and analysis in the social sciences and economics is often expensive, time-consuming, and notoriously difficult. The CBMS functions as a tool for evaluating poverty at the local level and enhancing accountability, transparency, and resource allocation by gathering data on LGU utilization [14].

### 2.2 Data descriptions and characteristics

27 variables totaling 426,600 (size) can be chosen for prediction out of 14,220 units with 240 attributes in the household dataset, which has a total size of 3,412,800. With a total size of 12,431,055, comprising 63,749 units and 195 attributes, 15 variables totaling 956,235 (size) can be chosen for prediction. The unprocessed datasets were meticulously organized and cleared. We painstakingly altered and coded them to comply with the econometric and machine learning presumptions and guidelines. The researchers performed advanced econometrics, produced prediction summaries, analyzed and statistically examined the data, and developed policies for economic growth. For the benefit of Filipinos' socioeconomic growth, Partido State University and University of the Philippines Los Baños have authorization to utilize, analyze, and share the datasets’ summaries, methods, and findings [1,15].

### 2.3 Indigenous communities data

Eight elements make up the dataset in the repository: population dynamics, indigenous people analytics, computational data on poverty, data dictionary, graph of results for poverty, table of poverty indices, multidimensional comprehensive data on poverty, and prescriptive policy analytics. The multidimensional poverty factors are included in an Excel file that includes 64 indicators from 8022 individuals from 12 tribes of the three sectors (Isarog, Ranggas, and Salog) and 1660 households of indigenous people [1].

### 2.4 Software requirements

The researchers have successfully and efficiently generated the essential data for economic development by using a variety of software programs, including MS Excel, SPSS, STATA, Python, and R. Aside from this, data that would be used as input for economic development was analyzed and relevant data was derived using diagnostic, prescriptive, predictive, and diagnostic models. Here are the necessary parameter tuning steps undertaken during the analysis.

1. Data Cleaning and Wrangling: This was performed using MS Excel, as it is an efficient tool for removing, deleting, and editing data contents.
2. Data Coding and Transformation: Data coding and transformation were carried out using MS Excel and STATA's replace commands.
3. Diagnostic Tests: Diagnostic tests were conducted using SPSS (Statistical Package for the Social Sciences) to determine whether the data meet statistical and econometric assumptions.
4. Econometric Modeling and Regression Analyses: Econometric modeling and regression analyses were performed using STATA and R software.
5. Machine Learning Regression and Classification: Machine learning regression and classification were implemented using R and Python.
6. Adjustment of Learning Rate: The learning rate was adjusted to control how quickly our model updates its parameters.
7. Modification of Regularization Parameters: Modifying the regularization parameters helps to manage overfitting by penalizing larger coefficients.
8. Feature Selection: Selecting the most relevant features can enhance model performance.
9. Cross-Validation: Utilizing k-fold cross-validation ensures a more robust evaluation of model performance.

Assumptions Made in Econometric Modeling and Machine Learning Analysis.

1. Linearity in Relationships: In regression analysis, we assumed that there is a linear relationship between the independent and dependent variables in probit or linear scale.
2. Independence of Residuals: Regression models assume that the residual errors are independent of one another. This assumption is vital for making valid statistical inferences.
3. Constant Variance of Residuals: The variance of the residuals should remain constant across all levels of the independent variables. If this assumption is violated, it may lead to inefficient estimates.
4. Normal Distribution of Residuals: For probit regression analyses, it is assumed that the residuals (errors) follow a normal distribution.
5. Absence of Multicollinearity: In regression analysis, the predictors should not be highly correlated with one another, as multicollinearity can result in unreliable coefficient estimates.
6. Exogeneity: It is assumed that the independent variables are not correlated with the error terms in regression models. Violation of this assumption can bias the model estimates.
7. Log Odds Linear Relationship: For logistic regression, it is assumed that the log odds of the dependent variable are linearly related to the independent variables.
8. Independence of Features: In machine learning classifiers, it is assumed that the features used for classification are independent of each other.
9. Separable Data Points in SVM: For methods such as Support Vector Machines (SVM), it is assumed that data points can be separated by a hyperplane. The distribution of data points may need to conform to certain geometric properties.
10. Correct Model Specification: It is assumed that the chosen model accurately captures the underlying process generating the data. Incorrect specifications can lead to biased predictions and interpretations.
11. Confusion Matrix: For classification models, utilize a confusion matrix to evaluate model accuracy by comparing the predicted classifications to the actual labels. It allows you to compute metrics like accuracy, precision, recall, and F1-score.
12. Adjusted R-squared: In regression analysis, calculate the adjusted R-squared to evaluate how well the model explains the variability of the dependent variable while penalizing for multiple predictors. This is particularly useful when comparing models with different numbers of predictors.

### 2.5 Indigenous people communities

Each of the twelve groups—which stood for the twelve tribes—formed the foundation for the multi-tribal analysis [1]. The researchers performed advanced econometrics, produced predicted summaries, acted as statisticians and data analysts to extract insights, and produced plans for economic growth. The datasets may be used and analyzed by Partido State University and University of the Philippines Los Baños, with permission to provide their instant summaries and conclusions for the socioeconomic progress of Filipinos.

### 2.6 Data indicators

All 64 variables are displayed at magnitude and proportion measurements along with descriptions in Table 1. When modeling and analyzing poverty using multidimensional dimensions, these variable sets are quite helpful. The community-based monitoring system (CBMS) was mined, wrangled, and clustered; an all-in system was used to fit the model; bidirectional, forward selection and backward elimination approaches were used; authors categorized the variables, and their work was reviewed; the local government provided a benchmark; the physical observation of poverty through in-person visits was also conducted; and data transparency and availability were taken into consideration.

**Table 1:** Variable codes and data descriptions that can be utilized for analytics and modeling.

Variable Codes	Data Descriptions
1A	Series of Households
2B	Series of Households per Tribe
1α	Name of Tribe
2Ξ	Tribal Cluster
3ξ	Total Household Members
4β	Male Household Members
5γ	Female Household Members
6Ι	Household with children under 5 years old
7ι	Total children under 5 years old
8δ	Child Malnutrition
9ε	Household with children aged 0–5 years old
10ζ	Total children aged 0–5 years old
11η	Child Mortality
12θ	Household with pregnant
13Κ	Household with death
14κ	Maternal Mortality
15λ	Informal Settlement
16Ο	Total Number of Informal settlers
17ο	Male Informal settlers
18μ	Female Informal settlers
19π	Makeshift Housing
20Π	Total Members in Makeshift Housing
21ρ	Male in Makeshift Housing
22φ	Female in Makeshift Housing
23χ	Access to safe drinking water
24ψ	Total Household members without access to safe drinking water
25Υ	Male members without access to safe drinking water
26υ	Female members without access to safe drinking water
27ω	Access to safe sanitary toilet facility
28α	Household members without access to sanitary toilet facility
29α	Male members without access to sanitary toilet facility
30Ξ	Female members without access to sanitary toilet facility
31ξ	Household with children for kindergarten
32β	Kindergarten Education
33γ	Household with members 6–11 years old
34Ι	Elementary Education
35ι	Household with members 12–15 years old
36δ	Junior High School Education
37ε	Household with members 16–17 years old
38ζ	Senior High School Education
39η	Poverty
40θ	Number of Poor Members
41Κ	Male Poor Members
42κ	Female Poor Members
43λ	Food Poverty
44Ο	Number of Poor Members
45ο	Male Food Poor Members
46μ	Female Food Poor Members
47π	Food Shortage
48Π	Total Members Suffered from Food shortage
49ρ	Male Members Suffered from Food shortage
50φ	Female Members Suffered from Food shortage
51χ	Total Labor Force in Household
52ψ	Male Members in Labor Force
53Υ	Female in Labor Force
54υ	Total Unemployed
55ω	Male Members Unemployed
56α	Female Members Unemployed
57α	Household with Labor force
58Ξ	Household with Unemployment
59ξ	Total Crimes
60β	Male members who are victims of crime
61γ	Female members who are victims of crime
62Ι	Household with Victims of crime
63ι	Dependency Ratio
64δ	Household Head Age

A variety of indicators drawn from frameworks for measuring multidimensional poverty are shown in Table 2. Researchers might simply use the collection of indicators given in Tables to replicate or re-implement the poverty measurements. Applying data analytics and econometrics techniques can result in a very useful table that quantifies the intangible and complex nature of poverty [1,[15], [16], [17], [18]].

**Table 2:** Indicator codes and data descriptions that can be utilized for poverty analytics and modeling.

Indicator Codes	Data Descriptions
65*N*	Series of Households per Tribe
66*T*	Tribe Code
67*PHH*	Poor Household
68*TPM*	Total Poor Members
69*TMPM*	Total Male Poor Members
70*TFPM*	Total Female Poor Members
71*FPH*	Food Poor Household
72*TMPM*	Total Male Poor Members
73*TFPM*	Total Female Poor Members
74*TPMF*	Total Food Poor Members
75z	Poverty Line
76*y*	Income Decile
77*g*	Gap
78*g/z*	Gap divided by Poverty Line
79*g/z*	Gap divided by Poverty Line validation
80sg*/z*	Squared gap divided by poverty line
81*z/y*	Poverty line divided by income decile
82*lnz/y*	Natural logarithm of Poverty line divided by income decile
83*lnz/y p*	Watts index per household
84H	Total Indigenous People Households
85N	Total Indigenous People Population

Together with the magnitude and proportion measurements of the seven main poverty categories, Table 3 compiles the 15 descriptors. These categories are crucial for describing and illustrating multidimensional poverty [15]. The codes are written in the old Filipino alphabet known as *Baybayin*, which was used by the country's indigenous inhabitants long before the contemporary English alphabet. The major need of indigenous populations determines the arrangement of the descriptors and underlying poverty groupings [3] (see Table 4).

**Table 3:** Underpinning Multidimensional Poverty Indicators at magnitude and percentage measures that are useful for modeling and analyzing indigenous socioeconomic conditions [15].

Code	Underpinning Poverty Indicators	Descriptions
	Earnings and Subsistence	Households earning less than the provincial poverty line
		Families whose income is below the food line
		Households that suffer from hunger
	Fundamental Education	Kids who are not enrolled in elementary school between the ages 6 to 11
		Children who are not enrolled in junior high school between the ages of 12 and 15
		Children who are do not attend senior high school between the ages of 16 and 17
	Employment	Households with members who are not employed in the workforce
	Nutrition and Well-Being	Children who died before the age of five
		Women who passed away from causes related to pregnancy
		undernourished children aged 0 to 5
	Harmony and Stability	Households affected of unlawful conduct
	Hygiene and Water	Households without access to clean drinking water
		Households without access to a hygienic sanitary restroom
	Residences and Land Settlement	Households residing in temporary housing
		households living in unofficial and unowned settlements

**Table 4:**
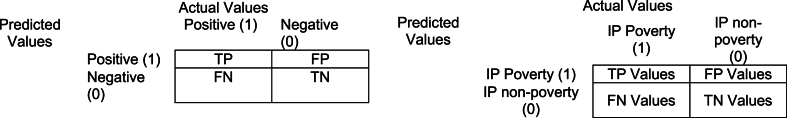
Indigenous people poverty prediction confusion matrix.

### 2.7 Empirical procedures

To look at the parameters of poverty and the results of indigenous people's development, a probit regression should be employed. Drawing from the models of various econometricians [3,[19], [20], [21], [22]], the researchers built and implemented the probit model in the following ways:

$$P\left(Y=1\;|X\right)=\Phi \;\left(X^{S}+\beta \right)$$

where: P stands for probability of being poor or not, Φ for the standard normal distribution's cumulative distribution function (the CDF), and β for the parameters under maximum likelihood estimate. The general model can be written as follows:

$$R\;\left(Y\left|X)=P(Y=1\right|X\right)=\Phi \;\left(\beta 0+\beta_{1}X\right)$$

A vector format was created by translating the model:

Y=Φ+Xβ+μ

Where:

Y = probit (p) = likelihood of a household being impoverished;

Φ is the standard normal distribution function cumulatively;

X = vector of independent variables;

β = vector of independent, control, and intervening variable coefficients, intercepts, or effects; and

μ stands for error term (see Table 5).

We utilize probit regression due to its effectiveness in modeling binary or dichotomous outcomes. In social science research involving indigenous populations, many phenomena are inherently binary in nature (see Table 6). Probit regression enables researchers to analyze and understand the relationships between predictor variables and the likelihood of these binary outcomes occurring. Furthermore, this method provides insights into how different factors impact the probability of an event occurring, offering a nuanced understanding of complex social phenomena. Its capability to handle nonlinear relationships and accommodate multiple independent variables makes probit regression a valuable tool for researchers aiming to uncover the intricate interplay of factors influencing social outcomes. We chose to apply probit regression because our dataset meets various econometric assumptions: linearity on the probit scale, independence, and the condition that the error term in the model follows a standard normal distribution. This assumption is crucial because probit regression models the cumulative distribution function of the standard normal distribution. Additionally, it satisfies conditions such as homoscedasticity, the absence of multicollinearity, exogeneity, and correct specification. Other scholars may be able to replicate the dataset descriptors for use in policymaking. They might employ the logit and probit econometric models, depending on the objectives of the study and the type of data they have. The modified and multiple fits of the logistic models can be used in a study to identify the features of variables that affect a person's poverty status [3,15,22,23]. The dependent variables are household poverty statuses based on food thresholds and income; the independent factors are multidimensional variables. Numerous intervening and controlling variables were also included in the models.

Y=α+Xβ+i+μ

Where:

**Table 5:** Population dynamics of indigenous people tribes in Southern Luzon, Philippines (2018–2020).

12 Tribes	Indigenous People HH	Total Number of HH	Distribution of IP HH	Indigenous People Population	Total Number of Population	Distribution of IP Population
Abucayan	102	515	19.8058	426	2041	20.8721
Balaynan	130	307	42.3453	656	1417	46.2950
Cagaycay	16	510	3.1373	67	2324	2.8830
Catagbacan	123	842	14.6081	603	3833	15.7318
Digdigon	115	639	17.9969	557	2980	18.6913
Hiwacloy	101	453	22.2958	451	2037	22.1404
Payatan	295	436	67.6606	1503	2189	68.6615
Pinaglabanan	153	468	32.6923	741	2290	32.3581
Salog	106	441	24.0363	478	1859	25.7127
San Isidro West	114	581	19.6213	564	2405	23.4511
San Pedro Aroro	287	320	89.6875	1308	1414	92.5035
Tabgon	118	429	27.5058	668	2252	29.6625
TOTAL	1660	5941	27.9414	8022	27,041	29.6661

**Table 6:** Multifaceted poverty and economic development indicators composed by the authors that can be utilized by the public for data analytics and policy initiatives.

Variables		VAR	Description	A Priori Expectation
Dependent Variables	Poverty Outcomes based on poverty line	*POPL*	1 (HH Living below Poverty Threshold), 0 (HH Not Living below Poverty Threshold)	
	Poverty Outcomes based on food threshold	*POFT*	1 (HH Living below Food Threshold), 0 (HH Not Living below Food Threshold)	
Independent Variables	Child Mortality	*CDEATH5*	1 (HH with Children under 5 who died), 0 (HH without Children under 5 who died)	Positive (+)
	Maternal Mortality	*WDEATHPC*	1 (HH with Women who died due to pregnancy related cases), 0 (HH without Women who died due to pregnancy related cases)	Negative (−)
	Malnutrition of Children	*CMALNO5*	1 (HH with children aged 0–5 who are malnourished), 0 (HH without children aged 0–5 who are malnourished)	Positive (+)
	Type of Housing	*MSHDWELL*	1 (HH who are living in Makeshift Housing), 0 (HH who are not living in Makeshift Housing)	Negative (−)
	Type of Settlement	*SQUATH*	1 (HH who are informal settlers), 0 (HH who are not living in Makeshift Housing)	Negative (−)
	Access to Safe Drinking Water	*WATACCESS*	1 (HH without Access to Safe Drinking Water), 0 (HH with Access to Safe Drinking Water)	Positive (+)
	Access to Sanitary Toilet Facility	*STFACCESS*	1 (HH without Access to Sanitary Toilet Facility), 0 (HH with Access to Sanitary Toilet Facility)	Positive (+)
	Children not attending Elementary	*CNAE*	1 (HH with children not attending elementary), 0 (HH with children attending elementary)	Positive (+)
	Children not attending Junior High School	*CNAJHS*	1 (HH with children not attending junior high school), 0 (HH with children attending junior high school)	Positive (+)
	Children not attending Senior High School	*CNASHS*	1 (HH with children not attending senior high school), 0 (HH with children attending senior high school)	Positive (+)
	Unemployment	*UNEMPL*	1 (HH with unemployment), 0 (HH without unemployment)	Negative (−)
	Victims of Crime	*VICCRIM*	1 (HH with victims of crime), 0 (HH without victims of crime)	Positive (+)
	Total Number of Household Members	*TNOHHM*	The total number of members of Indigenous People Households	Positive (+)
Interacting Variables	Type of Settlement and Total Number of Household Members	*SQUATH#TNOHHM*	Households who are informal settlers x Household Members	Positive (+)
	Type of Housing and Child Mortality	*MSHDWELL#CDEATH5*	Households living in makeshift housing x Children under 5 years old who died	Negative (−)
	Access to Safe Drinking Water and Child Mortality	*WATACCESS#CDEATH5*	Households without access to safe water x Children under 5 years old who died	Positive (+)
	Access to Safe Drinking Water and Total Number of Household Members	*WATACCESS#TNOHHM*	Households without access to safe water x Household Members	Negative (−)
	Access to Safe Drinking Water and Type of Settlement	*WATACCESS#SQUATH*	Households without access to safe water x Households who are informal settlers	Positive (+)
	Access to Safe Drinking Water and Type of Housing	*WATACCESS#MSHDWELL*	Households without access to safe water x Households living in makeshift housing	Positive (+)
	Access to Sanitary Toilet Facility and Type of Settlement	*STFACCESS# SQUATH*	Households without access to sanitary toilet facility x Households who are informal settlers	Negative (−)
	Access to Sanitary Toilet Facility and Type of Housing	*STFACCESS#MSHDWELL*	Households without access to sanitary toilet facility x Households living in makeshift housing	Positive (+)
	Children Malnutrition and Child Mortality	*CMALNO5#CDEATH5*	Malnourished children 0–5 years old x Children under 5 years old who died	Negative (−)

Y = logit (p) = log [p/(1-p)], where p is the likelihood that a household or an individual will be impoverished;

α represents the intercept, or individual impacts, of the following variables: housing and settlement, employment and livelihood, water and sanitation, health and nutrition, education, employment and order, and peace and order, which are taken to be constant;

X is the vector of independent variables, which includes control factors and is considered to be constant, such as socioeconomic conditions, education, health and nutrition, water and sanitation, housing and settlement, employment and livelihood, and peace and order;

β = vector of coefficients, intercepts, or effects on poverty outcomes that are considered to be constant from socioeconomic conditions, education, health and nutrition, water and sanitation, housing and settlement, employment and livelihood, and peace and order;

I = interfering factors or the combined effects of different socioeconomic circumstances, health and nutrition, education, housing and settlement, work and means of subsistence, water and sanitation, and peace and order; and

μ stands for error term.

Logistic regression is a popular choice in social science research for its versatility and robustness in modeling binary outcomes with multiple predictor variables. Unlike probit regression, logistic regression is more lenient in its assumptions while delivering comparable results. Its strength lies in its capability to estimate the probability of a binary event occurrence, accommodating various predictors effectively. Particularly in studies involving indigenous populations where non-linearity and non-normality are common, logistic regression proves invaluable. Its adaptability to handle both categorical and continuous variables, along with its interpretability, makes it a preferred tool for researchers aiming to predict and understand binary outcomes within the social sciences. Moreover, logistic regression can serve as a preliminary modeling step before delving into more complex predictive analytics methods in machine learning.The authors estimated the aforementioned models, which might be altered to include or exclude indicators unique to indigenous people that can be utilized for predictive analytics.

Model 1 *POPL* = β_0_ + β_1_*CDEATH5* + β_2_*WDEATHPC* + β_3_*CMALNO5* + β_4_ *MSHDWELL* + β_5_*SQUATH* + β_6_*WATACCESS* + β_7_*STFACCESS* + β_8_*CNAE* + β_9_*CNAJHS* + β_10_*CNASHS* + β_11_*UNEMPL* + β_12_*VICCRIM* + β_13_*TNOHHM* + β_14_*SQUATH#TNOHHM* + β_15_*MSHDWELL#CDEATH5* + β_16_*WATACCESS#CDEATH5* + β_17_*WATACCESS#TNOHHM* + β_18_*WATACCESS#SQUATH* + β_19_ *WATACCESS#MSHDWELL* + β_19_ *STFACCESS# SQUATH* + β_19_*STFACCESS#MSHDWELL* + β_19_*CMALNO5#CDEATH5* + μ
Model 2 *POFT* = β_0_ + β_1_*CDEATH5* + β_2_*WDEATHPC* + β_3_*CMALNO5* + β_4_ *MSHDWELL* + β_5_*SQUATH* + β_6_*WATACCESS* + β_7_*STFACCESS* + β_8_*CNAE* + β_9_*CNAJHS* + β_10_*CNASHS* + β_11_*UNEMPL* + β_12_*VICCRIM* + β_13_*TNOHHM* + β_14_*SQUATH#TNOHHM* + β_15_*MSHDWELL#CDEATH5* + β_16_*WATACCESS#CDEATH5* + β_17_*WATACCESS#TNOHHM* + β_18_*WATACCESS#SQUATH* + β_19_ *WATACCESS#MSHDWELL* + β_19_ *STFACCESS# SQUATH* + β_19_*STFACCESS#MSHDWELL* + β_19_*CMALNO5#CDEATH5* + μ

### 2.8 Poverty analytics

To evaluate the degree of poverty, the following metrics were put to use by Onsay (2022) [3]. These measurements may also be used by other researchers from around the globe to determine the poverty rate among their indigenous populations.

*W = Watts Index*; $W=\frac{1}{N}\;\sum_{i=1}^{N}\left[\ln\left(z\right)-\ln\left(y_{i}\right)\right]=\left(\frac{1}{N}\right)\;\sum_{i=1}^{q}\ln\left(\frac{z}{y_{i}}\right)$ where the total is divided among q persons whose income (yi) is less than the poverty line (z), and N population members are indexed in ascending order of income (or spending). The impoverished are added together, the poverty line is split by income, logs are taken, and the index is divided by the entire population. This is one of the first measurements of poverty that accounts for distribution [3,4]

$P_{0}$*= Headcount Ratio Po*$=\frac{1}{N}\;\sum_{i=1}^{N}\left(y_{i}<z\right)\text{;}$*Po*$=\frac{N_{P}}{N}$*Where, Np = Number of poor; and N = Total Population (or sample).* The headcount ratio (HCR) measures the proportion of the population that lives in poverty. The indicator function i returns 1 in the case of a true expression enclosed in brackets and 0 in the case of a false expression. As a result, if the household's income (yi) is less than the poverty line (z), where i = 1, it is considered to be poor. The main benefits of the headcount index are how simple it is to build and understand. The headcount ratio does, however, have a problem in that it fails to take into account the severity of poverty; that is, the headcount index remains constant as the poor get poorer [3,4].

$P_{1}=\frac{1}{N}\;\sum_{i=1}^{N}\frac{G_{i}}{z}$*Where,*$G_{i}$*= (z -*$x_{1})$ x $I\left(y_{i}<z\right)$*.* The poverty gap index is one way to gauge the level of poverty. It is defined as the population's average poverty gap expressed as a percentage of the poverty line, minus the impoverished, which is zero. It establishes the degree of poverty by looking at the average distance between the destitute and the poverty line. When the index approaches 1, the proportion of the population living in poverty rises, and when it falls below zero, the proportion of the population living in poverty decreases [3,4]

$P_{2}$ = Squared Poverty Gap Index; $P_{\propto }=\frac{1}{N}\;\sum_{i=1}^{N}{\left(\frac{G_{i}}{z}\right)}^{\propto },\left(\propto \geq 0\right)$ *Where* ∝ *=sensitivity of index to poverty; z=poverty line;* $x_{1}$ *=value of expenditure (income) per capita for ith person's HH; and* $G_{i}$ *= z -* $x_{1}$ *(with* $G_{i}=0\;when\;x_{i\;}>z$*) = poverty gap for individual i.* The poverty gap index is connected to the squared poverty gap index, also known as the poverty severity index. The poverty gap ratio's square is averaged to calculate it. The metric raises the weight given to an impoverished person's observed income as it goes below the poverty line by squaring each poverty gap data set. The squared poverty gap index is one type of weighted sum of poverty gaps where the weight is connected with the size of the gap [25].

### 2.9 Prediction methods

#### 2.9.1 Regression

1. CatBoost. Machine learning challenges requiring category, heterogeneous data are a good fit for CatBoost [26]. Its foundation is a gradient-boosted decision tree that may be applied to regression or classification tasks [[27], [28], [29]]. Here is how the authors modified the model: ${\hat{y}}^{poverty}=m\left(x\right)\sum_{i=1}^{Ntrees}\gamma \beta \;\left(x:d_{i}\right)$, where ${\hat{y}}^{poverty}$ is the target value; regression for prediction of poverty based on input attributes. The total number of trees is represented by Ntrees, and the learning rate, or γ, determines how much of Ntrees is used in the final prediction; $\beta \left(x:d_{i}\right)$, prediction of the i-th decision Ntrees in the ensemble, and $d_{i}$ represents the parameters of the i-th Ntrees.
2. eXtreme Gradient Boosting (XGBoost). This is one of the best supervised learning algorithms. The loss function is incorporated into the objective function and regularization, and the model contrasts the expected and actual values [30]. XGBoost is a scalable and effective gradient boosting framework that uses a number of regularization techniques, including column subsampling during tree construction, tree depth regularization, and L1 and L2 regularization on the leaf values, to control model complexity and prevent overfitting [31,32]. Here is how the authors modified the model: ${\hat{y}}^{poverty}=\sum_{h=1}^{S}m_{s}\left(x_{g}\right)=\sum_{h=1}^{S}v_{z}\left(x_{g}\right)$. Where ${\hat{y}}^{poverty}$ is the predicted value; $m_{s}\left(x_{g}\right)$ is the prediction of the s-th tree in the ensemble for the g-th households; $v_{z}\left(x_{g}\right)$ leaf weight z $\left(x_{g}\right)$ assigned to the leaf node corresponding to the g-th households; s total number of trees; z $\left(x_{g}\right)$ is the index of the leaf node for the i-th sample obtained by following the learned decision rules in s, and $x_{g}$ is the feature vector of the g-th household. XGBoost adds trees to the ensemble iteratively by using a gradient boosting technique. Every new tree is trained to adjust the prior ensemble's residuals. Hence, $o^{s}=\sum_{l=1}^{S}w\left(r_{l\;}\;{\hat{r}}_{l,}s-1\right)+\sum_{d=1}^{D}\Omega \left(s_{d}\right)$ where $o^{s}$ is the objective function being optimized for the s-th tree; $w\left(r_{l\;}\;{\hat{r}}_{l,}s-1\right)$ is the loss function that measures the difference between the true target value $r_{l\;}$ and the predicted value ${\hat{r}}_{l,}s-1$ by the ensemble s−1 trees; D is the maximum number of leaves in each tree; $s_{d}$ represents the d-th leaf in the s-th tree, and $\Omega \left(s_{d}\right)$ is the regularization term that penalizes the complexity of the tree, such as the tree depth or leaf weights.
3. Light Gradient Boosting Linear Machine (LightGBM). The node that maximizes the loss function's drop is divided. Our offering is a unique characteristic wrapping method that reduces basic explanations to just one feature [27,33,34]. In the following ways, the authors altered the model: ${\hat{y}}_{k}^{poverty}=\sum_{d=1}^{p}e_{d}a_{kd}+\omega$ where ${\hat{y}}_{k}^{poverty}$ is the predicted target value for the k-th households; $e_{d}$ represents the weight associated with the d-th feature; $a_{kd}$ is the value of the d-th feature for the k-th household; +ω is the bias term, and p is the total number of poverty features.
4. Linear Regression. This method applies the least squares methodology to fit models with continuous outputs. The lasso shrinkage method was used to reduce the prediction error [25,27]: ${\hat{\beta }}^{lasso}=\underset{\beta }{\underbrace{\mathrm{argmin}}}=\sum_{i=1}^{N}{\left(y_{i}-\beta_{0}-\sum_{j=1}^{p}x_{ij}\beta_{j}\right)}^{2}$. Subject to $\sum_{j=1}^{p}\left|\beta_{j}\right|\leq t$. The comparable Lagrange version displays the lasso penalty. $\sum_{i}^{p}\left|\beta_{j}\right|$. The solutions are nonlinear because of this constraint in the y_i_ [27,35]. The lasso selects subsets in a manner akin to continuous subset selection since some coefficients could be exactly zero when t is small. ${\hat{\beta }}^{lasso}=\underset{\beta }{\underbrace{\mathrm{argmin}}}\;\left\{\frac{1}{2}\sum_{i=1}^{N}{\left(y_{i}-\beta_{0}-\sum_{j=1}^{p}x_{ij}\beta_{j}\right)}^{2}+\lambda \;\sum_{j=1}^{p}\left|\beta_{j}\right|\leq t\right\}$.
5. Polynomial Regression. It is employed to minimize the discrepancy between the dependent variable's actual values and its anticipated values by identifying the ideal values for the coefficients that best suit the data [36,37]. In the following ways, the authors altered the model: ${\hat{y}}^{poverty}=\beta_{0}+\beta_{1}X+\beta_{2}X^{2}+\beta_{3}X^{3}+\beta_{4}X^{4}+\ldots \;\beta_{n}X^{n}$ where ${\hat{y}}^{poverty}$ is the predicted value of the dependent variable; X is the independent variable; β are the coefficients or parameters of the polynomial regression model, n is the degree of the polynomial, determining the highest power of x in the equation.
6. Random Forest. Schonlau and Zou (2020) claim that it finds explanatory variables and performs well. The Random Forest algorithm discovers nonlinear correlations between explanatory and dependent variables, making it one of the best machine learning techniques for determining the relevance of a variable [38]. The approach requires less hyperparameter tuning than conventional machine learning techniques [27,35,39]. In the following ways, the authors altered the model: The authors modified the model in the following ways: ${\hat{y}}^{poverty}=\frac{1}{2}\sum_{v=1}^{t}\gamma_{v}\;\left(c\right)$ where ${\hat{y}}^{poverty}$ is the predicted value of the target variable; t is the total number of decision trees in the Random Forest; $\gamma_{v}\;\left(c\right)$ represents the prediction of the v-th decision tree for the input features.
7. Support Vector Regression (SVR). It constructs an optimal separation hyperplane between two classes. Regression can be adjusted to use a continuous answer [27,35]. The authors modified the model in the following ways: ${\hat{y}}^{poverty}=\left(\;g,j\right)+q$ subject to $y_{k}$ $=\left(\;g,j_{k}\right)-q\leq \vartheta$; $\left(\;g,j_{k}\right)+q-y_{k}\leq \vartheta$ where ${\hat{y}}^{poverty}$ is the predicted value; j represents the input variables; g is the weight vector; q is the bias term; $y_{k}$ is the actual value of the target variable for the k-th sample; ϑ is the maximum allowable deviation or error; $y_{k}$ $=\left(\;g,j_{k}\right)-q\leq \vartheta$ is the first constraint ensures that the predicted value does not deviate more than ϑ from the actual value on the positive side; and $\left(\;g,j_{k}\right)+q-y_{k}\leq \vartheta$ is the second constraint ensures that the predicted value does not deviate more than ϑ from the actual value on the negative side.

#### 2.9.2 Classification algorithms

Various classification algorithms were devised and utilized to predict poverty outcomes of indigenous people.

1. *Adaptive Boosting (Adaboost)* creates a powerful classifier by combining multiple weak classifiers. It trains the weak classifiers iteratively, emphasizing cases that were incorrectly classified. To arrive at the final prediction, the predictions of all weak classifiers are merged and weighted based on their performance. AdaBoost is effective in handling difficult classification jobs and has applications in a wide range of fields [40]. The authors modified the model in the following ways: ${\hat{y}}^{poverty}=\sum_{t=1}^{T}\alpha_{t}\gamma_{t}\left(x\right)$, where x represents the input sample, y represents the vector of true labels for the training samples, and T represents the total number of weak classifiers used in AdaBoost. $\gamma_{t}\left(x\right)$ represents the vector of predictions of the t-th weak classifier for the N training samples. The weight $\alpha_{t}$ of the t-th weak classifier is calculated as: $\alpha_{t}=\frac{1}{2}\ln\left(\frac{1-\exists_{t}}{\exists_{t}}\right)$
2. *Decision Tree* is a machine learning technique that creates a tree-like model to separate input data into different classes or categories. It employs a hierarchical structure composed of internal and leaf nodes, where a judgment made in response to a particular aspect is reflected in each interior node and a class label is indicated by a leaf node. The method splits the input data recursively based on feature values and learns to maximize the separation of distinct classes. It selects the best feature and splitting criterion at each internal node to generate the most informative decision rules [41]. Each leaf node in the decision tree corresponds to a different class label. Let ∁ stand for the class label associated with a certain leaf node. Given an input vector x, the class label associated with the leaf node that results from executing the decision tree's path based on the input features is the prediction: ${\hat{y}}^{poverty}=∁$
3. *Gaussian Naïve Bayes* is an inexpensive yet effective method for classifying data using probabilities. Gaussian Naïve Bayes classification is a probabilistic method that relies on the assumptions of Gaussian distribution for continuous variables and feature independence for binary outcomes. It comprises figuring out each feature's variance and mean for each class in the training set. The method uses the Bayes theorem to calculate the posterior probability of each class given the observed feature values in order to classify a new instance. The highest posterior probability class is then assigned to ascertain the expected class [35,42]. This approach is easy to use and effective for binary classification tasks involving continuous features that could be represented by a Gaussian distribution. The authors modified the model in the following ways: ${\hat{y}}^{poverty}\left(C_{i}|x\;\right)=\frac{y\;\left(c_{i\;}\right)\;y\;\left(x|\;c_{i\;}\right)}{y\;\left(x\right)}$ where ${\hat{y}}^{poverty}\left(C_{i}|x\;\right)$ determines the posterior probability of each class given the observable features using the Bayes theorem.
4. *Gradient Boost* is a useful method for classifying binary data. It constructs a set of weak models that continually correct each other's mistakes in order to optimize an objective function. It has been shown that this approach is quite effective at producing accurate predictions about binary outcomes [43]. The authors modified the model in the following ways: ${\hat{y}}^{poverty}(y=\left(1|x\;\right)=\frac{1}{1+\exp-L\left(v\right)}$ where ${\hat{y}}^{poverty}(y=\left(1|x\;\right)$ translates the ensemble prediction into class probabilities using a sigmoid function. 1+exp−L(v) add the weighted prediction, v the total number of models in the ensemble, and the first to last prediction at log-odds of the target variable to represent the ensemble forecast.

*5. Extremely Randomized Trees (Extra Trees)* pertains to an ensemble learning strategy that uses decision trees. It divides nodes into several decision trees using random feature subsets and random thresholds. Compared to standard decision trees, Extra Trees lessen the chance of overfitting by increasing the randomness of the tree-building process. Extra Trees combine the predictions from many trees to produce robust and accurate predictions for both regression and classification applications [44]. Here is how we modified the model: ${\hat{y}}^{poverty}(y=\left(1|x\;\right)=\frac{1}{T}\sum_{t=1}^{T}\delta_{t}\left(x\right)$ where ${\hat{y}}^{poverty}(y=\left(1|x\;\right)$ δ_t (x) is the prediction on a current tree based on feature x of the input data; T is the total number of trees; this computation yields the average probability.

6. *Linear Discriminant Analysis (LDA)* a method of classification that uses linear combinations of features to separate data into discrete groupings. The data is projected onto a lower-dimensional space where class separability is maximized. LDA is helpful for classification problems because it makes an effort to reduce dimensionality while preserving the discriminating information between classes, especially when the classes are clearly divided [45].
7. *Logistic* is a useful method for solving binary classification issues. To model the relationship between the input features and the binary target variable, a logistic or sigmoid function is employed. When utilizing logistic regression, the computer estimates the likelihood that the target variable will fall into each class and produces predictions based on a preset threshold. Through an optimization process, logistic regression may classify new instances based on feature values by fitting the model to the training data and determining the coefficients for each feature [35].
8. *K-Nearest Neighbor* is a straightforward categorization method that determines the class label for a new instance by comparing its class labels with those of its nearest neighbors in the training data. It calculates the distance between each training instance and the new instance, selects the K nearest neighbors, and then utilizes a majority vote to decide on the class name. KNN doesn't make any assumptions about the distribution of the underlying data because it is non-parametric. Though it can change based on the distance metric and K that are selected, it is easy to understand and apply [46].
9. *Kernel SVM* is a powerful algorithm for binary classification that is also applicable to multi-class classification. Using a technique called the kernel trick, the input data is converted into a higher-dimensional feature space, and then the optimal hyperplane is found to maximally split the classes [47].
10. *Random Forest* performs admirably and identifies explanatory factors. The Random Forest algorithm, one of the best machine learning techniques for determining the significance of a variable, discovers nonlinear correlations between explanatory and dependent variables [38]. We modified the model in the following ways: ${\hat{y}}^{poverty}=\frac{1}{2}\sum_{v=1}^{t}\gamma_{v}\;\left(c\right)$ where ${\hat{y}}^{poverty}$ is the target variable's expected value; t is the Random Forest's total number of decision trees; $\gamma_{v}\;\left(c\right)$ reflects the input feature prediction made by the v-th decision tree. It is a popular ensemble learning method for tasks involving classification. To generate forecasts, it combines the individual outcomes of several decision trees. The ability of Random Forest can handle high-dimensional data, feature interactions, and noisy input is well known. It generates accurate and robust predictions while reducing the likelihood of overfitting. Additionally, it is capable of estimating feature importance, which offers information about the features that impact the categorization process the most.
11. *Support Vector Machine (SVR)* is an ideal separation hyperplane between two classes is built by it. ${\hat{y}}^{poverty}=\left(\;g,j\right)+q$ subject to $y_{k}$ $=\left(\;g,j_{k}\right)-q\leq \vartheta$; $\left(\;g,j_{k}\right)+q-y_{k}\leq \vartheta$ where ${\hat{y}}^{poverty}$ is the predicted value; j represents the input variables; g is the weight vector; q is the bias term; $y_{k}$ is the actual value of the target variable for the k-th sample; ϑ is the maximum allowable deviation or error; $y_{k}$ $=\left(\;g,j_{k}\right)-q\leq \vartheta$ The first constraint makes sure that the expected value doesn't differ from the actual value on the positive side by more than ϑ.; and $\left(\;g,j_{k}\right)+q-y_{k}\leq \vartheta$ The second constraint guarantees that there is no negative deviation of the projected value from the actual value of greater than ϑ. The algorithm is robust and finds the optimal hyperplane to separate the data into different classes. Support vector classification (SVC) locates the samples that are closest to the decision border or hyperplane during the training phase. It determines the optimal hyperplane by maximizing the margin, or the area between the support vectors and the hyperplane. SVC considers cases that are correctly classified as well as those that fall within a particular margin referred to as the soft margin in order to rectify certain misclassifications. When generating forecasts, SVC classifies new instances based on the edge of the decision boundary. The decision border separates the classes, and the confidence level of an instance is determined by its distance from the decision boundary. SVC is helpful for managing non-linearly separable data because it employs kernel functions to map the data into a higher-dimensional feature space. It is well known for its ability to handle high-dimensional data, resilience against noisy data, and ability to solve binary and multi-class classification issues [48,49]

#### 2.9.3 Indigenous people poverty prediction modeling

Data collection, preprocessing, econometric modeling and analysis, clustering, machine learning modeling and analysis, and performance evaluation are the six steps of our modeling method. We have replicated the experiences while implementing cross-validation using numerous training and test sets. We have also employed variance testing of the performance metrics and statistical significance tests.

a. *Data collection* – We collected the datasets through the community-based monitoring system in Goa, Camarines Sur.
b. *Data Pre-processing* – To satisfy econometric and machine learning assumptions, we have sorted, coded, and modified the datasets. For the first fitting of the model, we use a variety of transformations, feature scaling through normalization, and all-in, bidirectional, backward elimination, and forward selection.
c. *Econometric Modeling and Analysis* – To ascertain association and causation, 78 logistic and probit models for 34 localities, 4 sectors, and 1 municipal, were employed. The criteria that showed a substantial link with preparedness for catastrophe risk were given priority in machine learning prediction.
d*. Clustering* – Moreover, a few indicators needed by the classification system have been found by clustering. K-means clustering was used to separate the datasets into prepared and non-prepared categories. A technique was employed to accurately validate the input attributes because of the strong connection that existed between a number of the variables.

#### 2.9.4 Machine learning modeling and analysis

Eleven algorithms, known as regression and classification regressors, have been used to predict the outcomes of poverty for indigenous people. These operations were performed in two dimensions [50].

i. *IP Poverty prediction using a range of machine learning approaches*, we randomly picked training and test sets from the massive datasets in order to predict IP Poverty. The prediction strategies (generic R and Python techniques) were then described; and
ii. *IP Poverty prediction using pipeline (established criteria)* because we have constructed pipelines or other pre-defined criteria, we are able to apply the algorithms that will yield the best accuracy outcomes when it comes to IP Poverty prediction employing pipeline (existing criteria). We compare the accuracy results after the pipelines have been fitted to the training dataset. It was simpler to determine and predict which model was the most accurate based on the test dataset.

#### 2.9.5 Performance evaluation

We have examined each categorization algorithm's performance. Many assessment metrics were used for the classification process. We used the accuracy of classification, which is the ratio of the number of variables that were properly predicted to the total number of variables entered, $\alpha =\frac{\sigma }{\delta }$ , where α is the classification accuracy, σ is total number of correct predictions, and σ = is the total number of predictions made; The confusion matrix is an output matrix that displays the entire model's performance.

The matrix accuracy is $\alpha =\frac{\tau +\partial }{n}$ where α is the matrix accuracy, τ is the TP (Positive, positive prediction), ∂ is the FN (Positive, Negative predictions), and n is the total number of households; Precision $\rho =\frac{\tau }{\tau +\varphi }$ where ρ is the precision, τ is the TP (Positive, positive prediction), φ is the FP (Negative Positive predictions). It shows the correct positive results divided by the number of positive results divided by the classifier; Recall $∁=\frac{\tau }{\tau +\partial }$ where ∁ is the recall, τ is the TP (Positive, positive prediction), and ∂ is the FN (Positive, Negative predictions). It is the ratio of total correct positive results to all the relevant households; finally, the F1 = 2 $\left[\frac{1}{\left(\frac{1}{\frac{\tau }{\tau +\varphi }}\right)+\left(\frac{1}{\frac{\tau }{\tau +\partial }}\right)}\right]$. It reveals the test accuracy, simply the harmonic means between ρ and ∁. It tells how robust and precise the classifier is. Moreover, the scientific problems addressed in the article primarily aim to provide and present various methods for measuring the immeasurable welfare, poverty, and socioeconomic conditions and deprivations of indigenous peoples. In our comprehensive analysis of the sample's socioeconomic conditions, prediction and assessment of the multidimensional poverty situation, and the formulation and implementation of relevant policies through result analysis, we have detailed these aspects in a separate paper that focuses on results and policies linked to each finding. This paper can be found in the supplemental work. The work we present focuses on the methods used to generate these results. The policies outlined here serve as benchmarks for future researchers employing similar methods.

##### 2.9.5.1 Method application and validation

The table indicates that 11.84 % of all households in Goa, Camarines Sur are made up of Indigenous people, which equates to 12.58 % of the municipality's total population. These households do not represent a sample; instead, they encompass the entire population of indigenous people from the 12 tribes of Mt. Isarog (100 %). Although they are classified as indigenous peoples, they are also considered residents of our district, constituting 11.84 % of our total population (residents and indigenous people combined). In the Philippines, there are hundreds of different indigenous groups; however, our work focuses specifically on the 12 tribes of Mt. Isarog, known as Agta Tabangnon. The assumptions of generalizability, internal validity, and external validity regarding sample selection have been met by including the entire population of indigenous peoples unique to our mountains. Indigenous communities make up 27.94 % of the total households in the twelve tribes where they are found, with their combined participation accounting for 29.67 % of the overall population. San Pedro Aroro, Payatan, and Balaynan have the highest concentrations of indigenous people among the 12 barangays, with respective total population distributions of 92.50 %, 68.66 %, and 46.30 %. Cagaycay, Catagbacan, and Digdigon are the three least populated Indigenous People places out of 12, with a total population distribution of 2.88 %, 15.73 %, and 18.69 %, respectively [3]. This finding asserts that the population distribution of indigenous people is diverse [56], and that the CBMS is a useful tool for assessing population dynamics [58].

A portion of this work pertaining to policy proposals won grand champion in an international research competition (IRC) held in Asia, the variables that the authors produced had to be published as a prize in the competition proceedings. But the study only looks at health dynamics only [3]. Consequently, a great deal of information has not yet been adequately explored about a number of aspects, including peace and order, education, income and unemployment, and other interacting variables. Thus, more research will benefit greatly from the methods supplied in this paper because of their comprehensive analysis and production. The multidimensional poverty indicators that the authors have created and that the general public can use to promote economic development and reduce poverty are compiled in Table 1, Table 2 The poverty results at the income (z) threshold (poor based on a poverty line established by a particular region or nation) and the poverty outcomes at the food (f) threshold (poverty based on a food line set in a particular region or country) are the two dependent variables [15]. Nine (9) interaction factors and thirteen (13) multidimensional poverty indicators may be used as independent variables in the models. Based on econometric modeling, clustering, benchmarks from relevant literature, data accessibility, and the authors' diagnostic analytics, these indicators were selected.

**Fig. 1:**
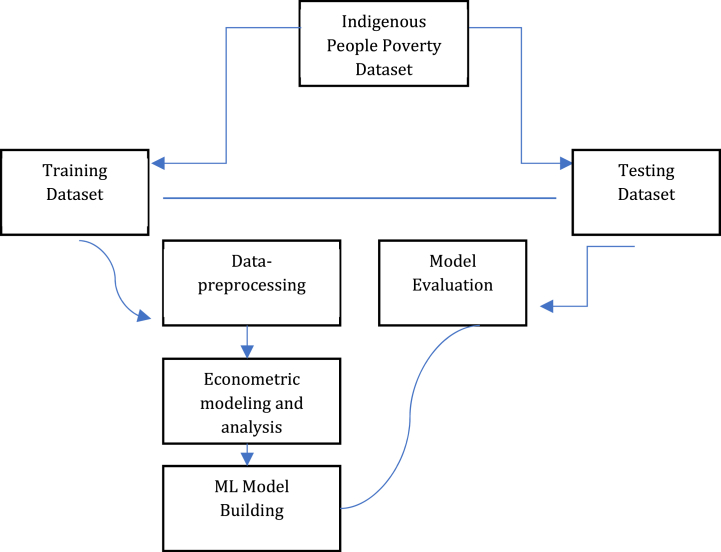
Indigenous people poverty prediction model displaying test and training data and the creation of a pipeline-based machine learning model.

The consolidated multidimensional socioeconomic and poverty profile of 12 Indigenous Tribes, comprising 1660 families and an overall Population of 8,022, is displayed in Table 7 based on the underpinning multidimensional variables shown in Table 3. These findings validate the usability of the CBMS data for poverty assessment [14], as it is important to evaluate the socioeconomic conditions of indigenous people to promote better welfare and address constraints within communities [3,6] (see Fig. 1).

**Table 7:** Multidimensional poverty and economic characteristics of indigenous people (Agta Tabangnon of 2018–2020).

Multidimensional Poverty and Economic Indicators		Household		Population	
		Magnitude	Proportion	Magnitude	Proportion
Earnings and Subsistence	Households earning less than the provincial poverty line	1428	86.02	7223	90.04
	Families whose income is below the food line	1230	74.10	6301	78.55
	Households that suffer from hunger	17	1.02	102	1.27
Fundamental Education	Kids who are not enrolled in elementary school between the ages 6 to 11 ∗Total # of HH with children aged 6–11 = 831 ∗Total population of children aged 6–11 years old = 1430	278	33.45	562	39.30
	Children who are not enrolled in junior high school between the ages of 12 and 15 ∗Total # of HH with children aged 12–15 years old = 606 ∗Total population of children aged 12–15 years old = 854	342	56.44	529	61.94
	Children who are do not attend senior high school between the ages of 16 and 17 ∗Total # of HH with children aged 16–17 = 331 ∗Total population of children aged 16–17 = 352	271	81.87	288	81.82
Employment	Unemployed members of the labor force ∗Total # of HH with members of the labor force = 1423 ∗Total population of members of the labor force = 1979	44	3.09	46	2.32
Nutrition and Well-Being	Children who died before the age of five ∗Total HH with children under 5 years old = 757 ∗Total population of children under 5 years old = 1066	11	1.45	11	1.03
	Women who passed away from causes related to pregnancy	0	0.00	0	0.00
	Undernourished children aged 0 to 5 ∗Total number of children 0–5 years old = 843 ∗Total population of children aged 0–5 years old = 1297	104	12.34	109	8.40
Harmony and Stability	Households affected of unlawful conduct	14	0.84	14	0.17
Hygiene and Water	Households without access to clean drinking water	374	22.53	1863	23.22
	Households without access to a hygienic sanitary restroom	448	26.99	2068	25.78
Residences and Land Settlement	Households residing in temporary housing	80	4.82	381	4.75
	households living in unofficial and unowned settlements	183	11.02	904	11.27

**Fig. 2:**
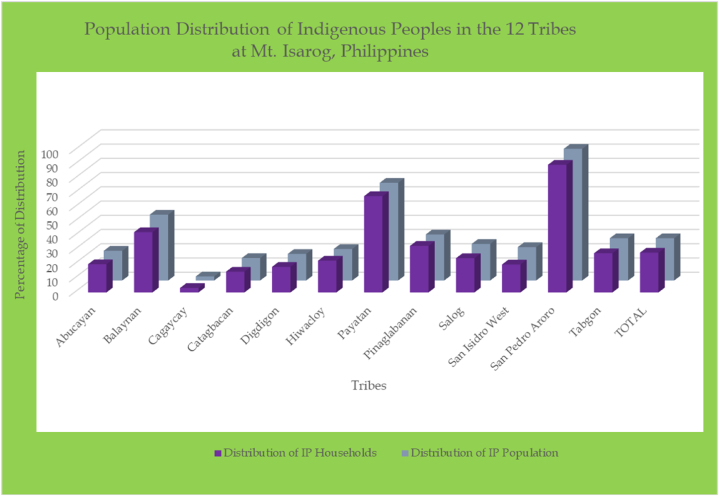
Population distribution of Indigenous Peoples in the 12 tribes of Mt. Isarog, Philippines.

**Fig. 3:**
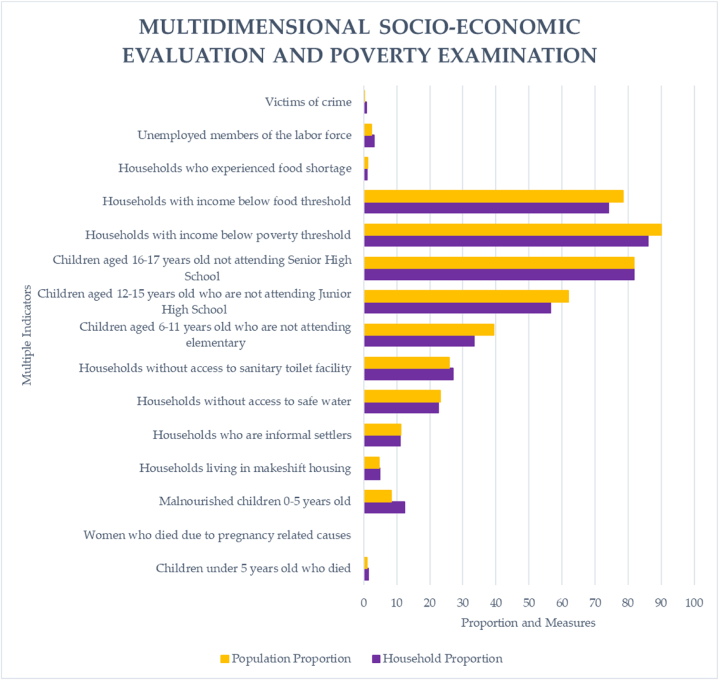
Multidimensional socioeconomic evaluation and poverty examination at households and individual measurements.

Fig. 2 shows multidimensional socio-economic evaluation and poverty examination of our indigenous people at households and individual measurements [15]. Eighty-two percent of people live in poverty, and seventy-one percent do not have access to food. There have been some reports of unemployment and food scarcity, but not much. Fourteen cases of crimes against indigenous people were reported during the census period. Regarding health and nutrition, 1.45 % of all homes with children under five have experienced a death or malnourishment, and 12.34 % of all households with children between the ages of 0 and 5 have experienced both. Merely 4.82 % of all households and 11.02 % of informal settlers, respectively, reside in temporary dwellings. The statistics show that just 22.53 % and 26.99 % of all families, respectively, have access to sanitary restrooms and safe drinking water. The majority of children in the community are either not enrolled in school or have stopped enrolment as educational levels rise, according to evaluation results that are largely consistent across barangays. Based on the results, it can be inferred that the main factors causing poverty among indigenous people in 12 tribes are sources of income and livelihood as well as a basic education. Furthermore, poverty has been shown to affect housing, access to clean water and sanitation, health and nutrition, and other aspects of daily life that have an impact on welfare. These findings reject the null hypothesis that multidimensional socioeconomic deprivations do not influence poverty outcomes. They assert, supported by various studies, that the multifaceted characteristics of households influence the incidence of poverty, particularly in indigenous communities [3,4,15,23,24,59] (see Fig. 3).

According to the data on poverty incidence, Salog and Catagbacan have the fewest impoverished homes among the 12 tribes, while Abucayan is the poorest area, followed by Pinaglabanan and Payatan. Indigenous people experience moderate to severe poverty, according to the poverty gap. Payatan and Balaynan are the next two places with the lowest poverty gap index, after Abucayan. However, compared to other barangays, Salog and San Pedro Aroro have the lowest poverty gap score, indicating higher incomes. Based on severity statistics, the tribes of Abucayan, Balaynan, Payatan, and Pinaglabanan have more extreme poverty. However, Salog, Hiwacloy, and San Pedro Aroro experience less severity. By using the Watts Index decomposition, the results demonstrate that poverty is more extensive in Abucayan, Balaynan, Digdigon, Payatan, and Tabgon, but less prevalent in Salog, Hiwacloy, San Pedro Aroro, and Cagaycay. This analysis asserts that poverty is multidimensional and has many faces [3,4].

A regression sample was run and Table 8 illustrates the results. The prediction equation is defined as log(*p*/1-*p*) = β_0_ + β_1_*CDEATH5* + β_2_*WDEATHPC* + β_3_C*MALNO5* + β_4_*MSHDWELL* + β_5_*SQUATH* + β_6_*WATACCESS* + β_7_*STFACCESS* + β_8_*TNOHHM* + μ*.* where *p* is the probability of poverty outcomes. Only three indicators—household members, households without access to safe water, and informal settlers—significantly predict the poverty outcome variable, according to the findings. Table 8 provides some structures and indicators that can be utilized. Researchers can duplicate and reproduce the models by changing the variables according to their intended use.

**Table 8:** Results of non-linear regression on poverty classification of indigenous people.

Poverty Class	Odds Ratio	Coefficient	Standard Error	z	p>|z|	[95 % Conf. Interval]	
Malnourished children 0–5 years old	2.0381	0.4285	0.9676	1.5000	0.1340	0.8038	5.1679
Children under 5 years old who died	0.9359	−0.1282	1.0259	−0.0600	0.9520	0.1092	8.0220
Women who died due to pregnancy related causes	1.0000	0.0000	0.0000	0.0000	0.0000	0.0000	0.0000
Households who are informal settlers	1.6839	1.4063	0.4609	1.9000	0.0570	0.9847	2.8795
Households living in makeshift housing	0.9390	0.3991	0.3274	−0.1800	0.8570	0.4741	1.8595
Households without access to safe water	1.7541	0.3200	0.3791	2.6000	0.0090	1.1485	2.6792
Households without access to sanitary toilet facility	1.3992	0.4204	0.2629	1.7900	0.0740	0.9681	2.0223
Household Members	1.4608	0.3864	0.0623	8.8800	0.0000	1.3436	1.5882
Households who are informal settlers x Household Members	0.8151	−0.2078	0.1160	−1.4400	0.1510	0.6167	1.0774
Households without access to safe water x Household Members	1.0785	0.0756	0.1311	0.6200	0.5340	0.8499	1.3687
Households without access to safe water x Households who are informal settlers	0.7321	−0.3118	0.6753	−0.3400	0.7350	0.1201	4.4643
Households without access to safe water x Households living in makeshift housing	0.7095	−0.3433	0.6108	−0.4000	0.6900	0.1313	3.8345
Households without access to sanitary toilet facility x Households who are informal settlers	1.0910	0.0871	0.9926	0.1000	0.9240	0.1834	6.4897
Households without access to sanitary toilet facility x Households living in makeshift housing	0.3326	−1.1010	0.2518	−1.4500	0.1460	0.0754	1.4669
Constant	0.9408	−0.0611	0.1943	−0.3000	0.7670	0.6276	1.4102

**Fig. 4:**
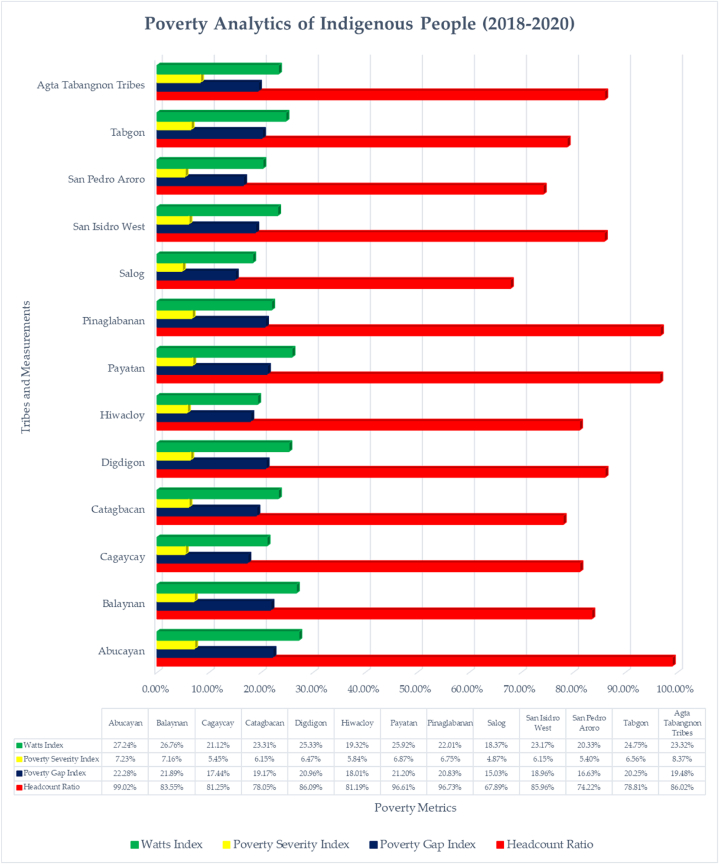
Poverty analytics of Indigenous People in Southern Luzon, Philippines, that scholars studying Indigenous people in other parts of the world may duplicate.

The likelihood of informal settlers living below the poverty level is higher than that of formal settlers. Moreover, the probability of poverty increases with the number of household members. The least likely to be below the poverty line are households with fewer people and households having access to sanitary restrooms. According to the findings, one important predictor of poverty is the number of household members which is being displayed by Fig. 4. These results reject the null hypothesis that family size does not influence poverty outcomes. This concurs with various studies conducted on families in the Philippines [3,4,15,23,24,59].

The results of classification statistics on the econometric models that researchers employ are displayed in Table 9. There are 225 non-poor samples and 1398 poor samples. The model properly classifies all 1623 observations, indicating 100 % sensitivity. Therefore, the model accurately predicted every home below the poverty line. Overall, 86.14 % of cases were appropriately classified. Consequently, the home observations in the logistic model are accurately classified by the models or alternative specifications. It is possible to create more modeling and alter the categorization statistics.

**Table 9:** Classification statistics of econometric models utilized by researchers.

Classifications	Poor	Non-Poor	Total
+	1398	225	1623
–	0	0	0
Total	1398	225	1623
Sensitivity			100.00 %
Specificity			0.00 %
Positive predictive value			86.14 %
Negative predictive value			.%
False +			100.00 %
False -			0.00 %
False + rate for classified +			13.86 %
False - rate for classified -			.%
Correctly classified			86.14 %

**Fig. 5:**
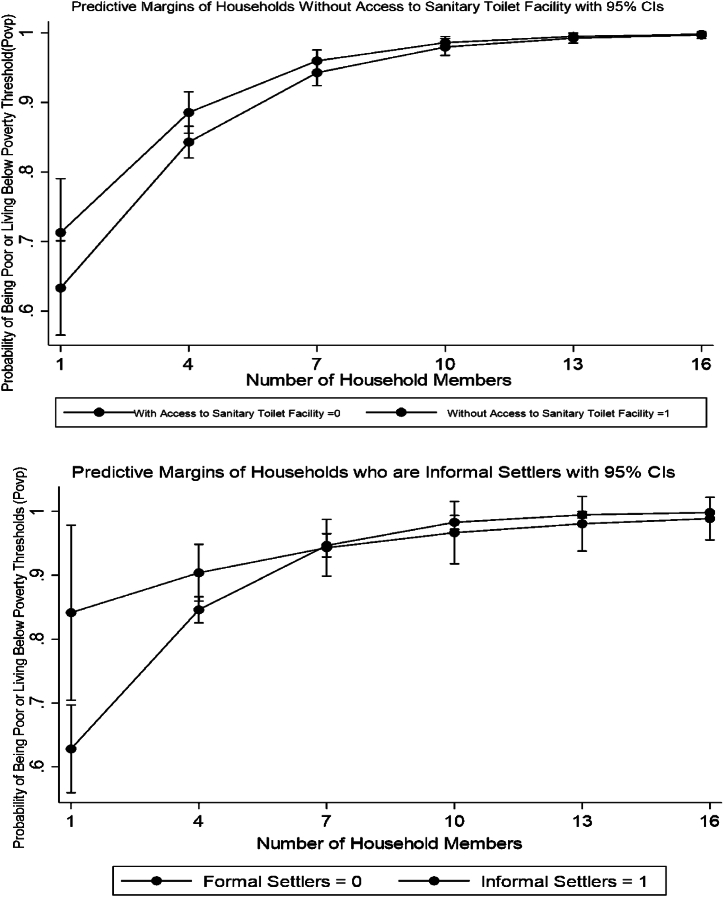
Predictive analytics demonstrating predictive margins on poverty outcomes that academics studying Indigenous people in different parts of the world can duplicate.

We have put the processes into practice and used cross-validation and prudence when applying all machine learning algorithms. Fig. 5 depicts the average mean square error of machine learning regressors in poverty prediction for indigenous people tribes. Regression analysis uses poverty gap and severity statistics to support the target value, which is the Watts Index decomposition. In this work, seven regressors are used: Random Forest, Polynomial, SVR, XGBoost, LightGBM, Linear, and CatBoost. The accuracy of the machine learning models is guaranteed by disaggregating the data at different configurations. Different ensembles are helpful in this kind of study since different localities have different properties. The machine learning algorithms that we have employed are largely designed to support policies and programs for local sectors and microeconomic views. These results align with the assumptions of algorithms used to generate reliable outcomes for indigenous people [[26], [27], [28], [29], [30], [31], [32], [33], [34], [35], [36], [37], [38], [39],^59^].

**Fig. 6:**
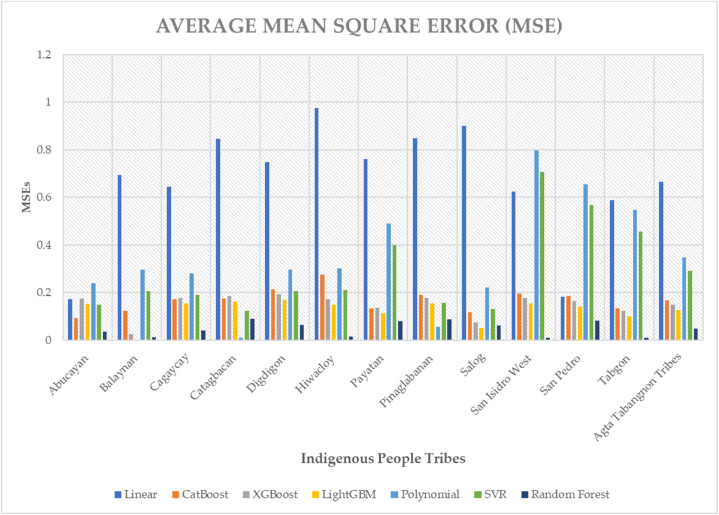
Average mean square error of machine learning regressors in poverty prediction for indigenous people tribes.

Twelve tribes have 91 runs of poverty forecasts at disaggregation, which are then pooled. The average mean square error of the 12 tribes' machine learning regressors is shown in Fig. 5 and the average R-square of machine learning regressors is displayed in Fig. 6. The analysis's findings show that, out of all the tribes, the random forest regression technique has the lowest mean square error—0.5497—behind LightGBM and XGBoost. With an R-square of 0.4222, linear regression has the lowest R-square of any method. An SVR and a polynomial algorithm come next. The random forest regressor yielded the greatest R-square, 0.9208, whereas CatBoost, XGBoost, and LightGBM all shown comparable performance. It suggests that the regression that best matches the regression line and has the lowest error score is the random forest regression [59].

**Fig. 7:**
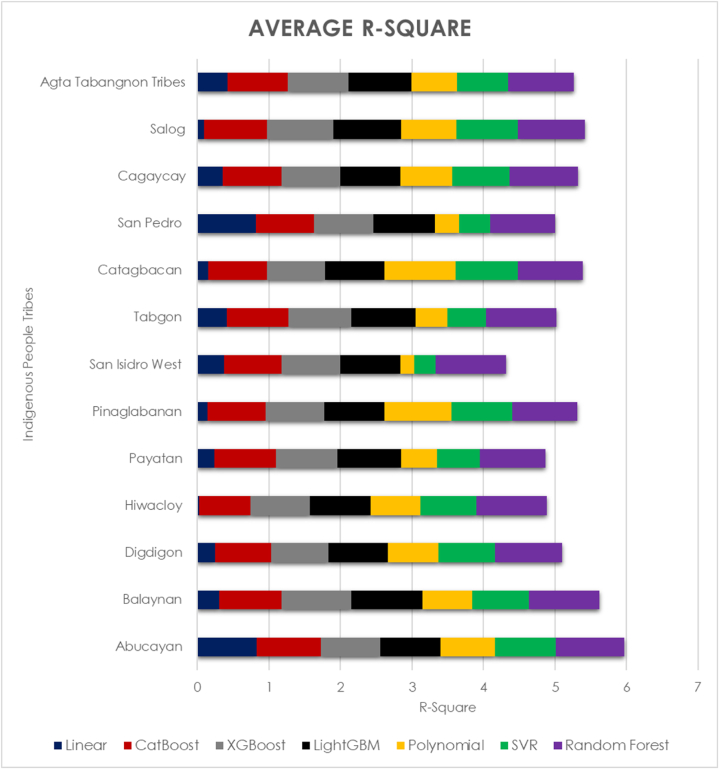
Average R-square of machine learning regressors in poverty prediction for indigenous people tribes.

**Fig. 8:**
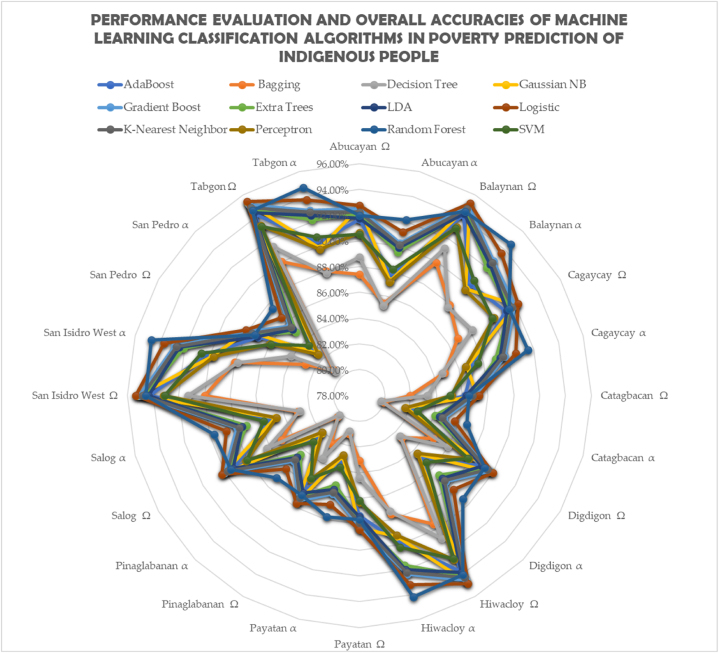
Performance comparison and evaluation of overall accuracies of machine learning classifiers in poverty prediction for indigenous people tribes.

The target value for categorization analysis is the incidence of poverty and the poverty outcomes that econometric models support. AdaBoost, Bagging, Decision Tree, Gaussian Naïve Bayes, Gradient Boost, Extra Trees, LDA, Logistic, K-Nearest Neighbor, Perceptron, Random Forest, and Support Vector Machine are the twelve classifiers used in this work. Fig. 7 reveals the performance comparison and evaluation of overall accuracies of machine learning classifiers in poverty prediction for indigenous people tribes. Twelve tribes were formed by clustering the datasets. Optimizing and guaranteeing the correctness of the ML model classifiers is achieved by disaggregating the data at different configurations. Different ensembles are helpful in this kind of study since different localities have different properties. Twelve tribes have 156 runs of poverty projections, which are then combined. The performance comparison of machine learning classifiers for 12 tribes at random and pipelines is shown in Table 3. The random forest regression classifier has the best accuracy of 90.69 % for all tribes, according to the analysis's results; it is followed by K-Nearest Neighbor, Gradient Boost, and Logistic. Baggin and Decision Tree algorithms have the lowest accuracy. Similar findings were obtained with pipeline algorithms. On the other hand, classifier accuracy rates have gone up. With 94.89 % accuracy, the random forest classification has the highest rate. Logistic classification and gradient boost classification come next [59]. Fig. 6 shows the performance evaluation of all machine learning classifier accuracies for 12 tribes at random pipelines and states (see Fig. 8).

Targeting policies for programs prioritized for economic development will be facilitated by the policy mapping presented in Table 10. This map was created using health dynamics of indigenous people expressed as a percentage of the total population of all tribes. The processes of grouping and ranking were completed using R. Due to the inclusion of twelve tribes in the study, they were divided into two groups: high prevalence and low prevalence. This classification meant that the six tribes with the highest proportions were categorized as having high prevalence, while the six with the lowest proportions were categorized as having low prevalence. This system simplified the process of setting priorities for initiatives and directing policies effectively. Other researchers studying indigenous people can replicate these results and may also include additional variables not depicted in the figures [3]. The work employs policy mapping to illustrate the concept of health dynamics. Using predictive analytics and the available databases, researchers from around the world may be able to map policies for our indigenous people—not just in the realm of health but beyond.

**Table 10:** A scenario of a policy mapping for economic development.

Tribes	Child Mortality	Pregnancy Mortality	Malnutrition of Children	Makeshift Shelters	Informal Settlement	Access and Supply of Safe Water	Access to sanitary toilet facility
Abucayan	–	–	–	(6)↑	(6)↑	(2)↑	(6)↑
Balaynan	(5)↑	–	(4)↑	(8)↓	(4)↑	(4)↑	(8)↓
Cagaycay	–	–	(6)↑	(3)↑	(2)↑	(6)↑	(9)↓
Catagbacan	–	–	–	(7)↓	(10)↓	(5)↑	(2)↑
Digdigon	(4)↑	–	(8)↓	(4)↑	(9)↓	(9)↓	(3)↑
Hiwacloy	–	–	(7)↓	(10)↓	(5)↑	(1)↑	(1)↑
Payatan	(3)↑	–	(1)↑	(12)↓	(3)↑	(10)↓	(5)↑
Pinaglabanan	(2)↑	–	(9)↓	(11)↓	(1)↑	(7)↓	(11)↓
Salog	–	–	(5)↑	(9)↓	(8)↓	(11)↓	(12)↓
San Isidro West	(1)↑	–	(2)↑	(1)↑	(7)↓	(8)↓	(10)↓
San Pedro Aroro	(6)↑	–	(3)↑	(2)↑	(11)↓	(12)↓	(7)↓
Tabgon	–	–	(10)↓	(5)↑	(12)↓	(3)↑	(4)↑

Legend: (No Entry); ↑ (High degree of prevalence); ↓ (Low degree of prevalence); and (1) Rank.

Based on policy mapping and predictive analytics methods, Table 11 presents a summary of the outcomes for policy targeting derived from predictive analytics. Numerous potential intervention programs, legislative efforts, and economic development projects are being proposed for each multidimensional socioeconomic and poverty indicator [3,59]. By examining the table, policy targeting has become feasible. These strategies would be extremely beneficial to the tribes that need to prioritize specific issues or aspects. Ultimately, indigenous people will not be left behind—their poverty conditions will be alleviated, and their economy will be developed.

**Table 11:** Summary of predictive analytics results for policy targeting.

Poverty Indicators		Possible Intervention Programs/Policy Initiatives/proposed projects for Economic Development	Priority Tribes
**Health and Nutrition**	Child Mortality	• Track and Monitor children with sickness or illness • Provide Trainings on Proper Nutrition and Food preparation • Information Education and Communication (IEC) Campaigns and Materials on proper hygiene, health and sanitation • Continuous home visit of health workers and regular check-up and supplementation for children age 0–5 years old • Food Nutrition Program for Combatting Malnutrition • Introduction of Household-based food production • Legislation/ordinances/impact monitoring to lessen vulnerabilities and reduce mortality in the locality	➢ Balaynan ➢ Digdigon ➢ Payatan ➢ Pinaglabanan ➢ San Isidro West ➢ San Pedro Aroro
	Pregnancy or Maternal Mortality	• Strict monitoring of pregnant women for their regular check-ups and vaccination • Strict implementation of the Municipal Ordinance on Facility-Based Delivery (prohibiting home delivery) • Goal setting of completing 4 ANC	➢ San Isidro West ➢ San Pedro Aroro ➢ Payatan ➢ Payatan ➢ Pinaglabanan ➢ Cagaycay
	Malnutrition of Children	• Feeding program • Mother's Class • Health check-ups and supplements • Health Counselling • Program-based food production(backyard organic gardening backed up with local legislation/ordinances/impact monitoring	➢ Balaynan ➢ Cagaycay ➢ Payatan ➢ Salog ➢ San Isidro West ➢ San Pedro Aroro
**Housing**	Makeshift Shelters	• Identify a potential relocation site for housing projects with livelihood programs and access to essential services. • Provision of Housing Units to informal settlers • Rent-to-own housing projects scheme in coordination with HUDCC and other lined agencies for eligible beneficiaries. • Adoption and strict enforcement of Barangay Zoning Ordinance	➢ Abucayan ➢ Cagaycay ➢ Digdigon ➢ San Isidro West ➢ San Pedro Aroro ➢ Tabgon
	Informal Settlement		➢ Abucayan ➢ Balaynan ➢ Cagaycay ➢ Hiwacloy ➢ Payatan ➢ Pinaglabanan
**Water and Sanitation**	Access and Supply of Safe Water	• Conduct watershed development training • Information Education and Communication (IEC) on Health and Sanitation • Water testing and treatment of existing water sources, • Provision of Safe water facility.	➢ Abucayan ➢ Balaynan ➢ Cagaycay ➢ Catagbacan ➢ Hiwacloy ➢ Tabgon
	Access to sanitary toilet facility	• Provision of sanitary toilets. • Strict implementation of Ordinance on Health and Sanitation	➢ Abucayan ➢ Catagbacan ➢ Digdigon ➢ Payatan ➢ Tabgon
**Basic Education**	Children aged 6–11 years old who are not attending elementary	• Feeding programs for Children • Provision of School supplies and uniforms to identified children age 6–11 not attending elementary • Implementation of Ordinances for the members of the 4Ps • Information Education and Communication (IEC) on literacy campaign • Ordinances/local legislation/policies • Various support to teachers and learners • Partnership with academic and private institutions for education campaigns and extension programs	➢ Abucayan ➢ Catagbacan ➢ Digdigon ➢ Pinaglabanan ➢ San Isidro West ➢ Tabgon
	Children aged 12–15 years old who are not attending Junior High School	• ALS Program • TESDA Skills training • Policies/Ordinances • Various support to teachers and learners • Partnership with academic and private institutions for education campaigns and extension programs	➢ Catagbacan ➢ Digdigon ➢ Abucayan ➢ Pinaglabanan ➢ San Isidro West ➢ Tabgon
	Children aged 16–17 years old not attending Senior High School	• ALS Program, • TESDA Skills training • Policies/Ordinances • Various support to teachers and learners • Partnership with academic and private institutions for education campaigns and extension programs	➢ Abucayan ➢ Pinaglabanan ➢ San Isidro West ➢ Catagbacan ➢ Digdigon ➢ Tabgon
**Income and Livelihood**	Poverty based on Income Threshold	• Backyard gardening • Proper technology on farming and impact monitoring of programs and projects (DA) • Ordinance on backyard gardening • Women empowerment (Livelihood programs) • Technical Skills training (TESDA) • Advocacy training on Food preparation/processing • Ordinances/local legislation/policies • Partnership with academic and private institutions for skills training and extension programs	➢ Abucayan ➢ Payatan ➢ Pinaglabanan ➢ Balaynan ➢ Digdigon ➢ San Isidro West
	Poverty based on Food Threshold		➢ Abucayan ➢ Payatan ➢ Pinaglabanan ➢ Balaynan ➢ Digdigon ➢ San Isidro West
	Food Shortage		➢ Abucayan ➢ Payatan ➢ Pinaglabanan ➢ San Isidro West ➢ Balaynan ➢ Digdigon
	Unemployment		➢ Pinaglabanan ➢ San Isidro West ➢ Balaynan ➢ Digdigon ➢ Abucayan ➢ Payatan
**Peace and Order**	Victims of crime	• CCTV (high definition with registered plate recognition) • Profiling of all the migrants to our municipality within five years • Strict implementation of Curfew hours • Local Ordinance on Excessive use of gadgets and social media	➢ Payatan ➢ Salog ➢ San Pedro Aroro ➢ Digdigon ➢ Balaynan ➢ Catagbacan

## 3 Method discussions

The representation of Indigenous peoples in the municipality emphasizes their integral role within the community. This demographic data can inform local policies and community programs aimed at supporting Indigenous rights and development. The significant disparities in population concentrations among barangays suggest varying levels of integration and resources available to Indigenous communities. Factors contributing to these disparities should be explored to understand their implications for resource allocation and community support. Moreover, the methodology of including the entire population of Indigenous peoples enhances the robustness of the findings, allowing for a more comprehensive understanding of Indigenous demographics compared to studies relying on smaller, sampled populations. The study focuses on multidimensional factors affecting indigenous communities as we acknowledge that many critical aspects, such as peace and order, education, income, unemployment, and other interacting variables, have not been sufficiently explored. This gap suggests that further research would greatly benefit from the comprehensive methods outlined in this paper, which can facilitate a deeper understanding of these interconnected issues. We have developed multidimensional poverty indicators that are essential for promoting economic development and reducing poverty. The findings validate the utility of the Community-Based Monitoring System (CBMS) data in assessing poverty, underscoring the importance of evaluating the socioeconomic conditions of Indigenous peoples. Such assessments are critical for promoting better welfare and addressing the constraints faced within these communities. This comprehensive approach not only enhances our understanding of poverty dynamics but also informs targeted interventions that can lead to sustainable development for Indigenous populations. It can be inferred that the principal factors driving poverty among the Indigenous peoples of the 12 tribes are directly related to income sources, livelihood opportunities, and access to basic education.

The methods and data illustrate that poverty impacts various facets of daily life, including housing quality, access to clean water and sanitation, and overall health and nutrition. This multifaceted nature of poverty challenges the null hypothesis that multidimensional socio-economic deprivations do not influence poverty outcomes. Instead, our results affirm that the intersecting characteristics of households—such as income, education, and access to services—significantly influence poverty incidence, particularly within Indigenous communities. The poverty incidence among the 12 tribes reveals significant disparities, with Salog and Catagbacan having the fewest impoverished homes, while Abucayan is identified as the poorest area, followed by Pinaglabanan and Payatan. The analysis indicates that Indigenous peoples experience moderate to severe poverty, particularly in Abucayan, Balaynan, and Payatan, which exhibit high poverty severity. Regression analysis shows that household size, lack of access to safe water, and informal settlement status are significant predictors of poverty outcomes, with larger households more likely to experience poverty. Additionally, the study employs various machine learning algorithms, including Random Forest and XGBoost, to predict poverty outcomes, as illustrated by the average mean square error results. These models effectively identify poverty levels and can inform targeted policies and programs to address the unique challenges faced by Indigenous communities. The findings reinforce the multidimensional nature of poverty and highlight the importance of data-driven approaches in developing effective interventions for improving welfare in these populations.The analysis of poverty forecasts across the twelve tribes demonstrates the effective use of machine learning techniques to assess poverty outcomes. Among the various methods employed, the random forest regression technique emerged as the most reliable, showing superior performance in fitting the data and minimizing prediction errors. This indicates that random forest is particularly adept at capturing the complexities of poverty dynamics within these communities. In addition to regression analysis, a range of classifiers, including AdaBoost, Decision Tree, and Support Vector Machine, were utilized to categorize poverty incidence. The results highlight that random forest consistently achieved the highest accuracy in predicting poverty across the tribes, followed closely by other classifiers like K-Nearest Neighbor and Gradient Boost. This strong performance underscores the importance of using machine learning models tailored to local contexts, as different areas may exhibit unique characteristics influencing poverty. Overall, these findings illustrate the potential of machine learning to inform targeted interventions and policies aimed at alleviating poverty in Indigenous communities, reinforcing the need for data-driven strategies in addressing their specific challenges.

The policy mapping presented in the study serves as a crucial tool for prioritizing economic development initiatives tailored to Indigenous communities. By analyzing health dynamics as a percentage of the total population across the twelve tribes, the tribes were classified into two distinct groups: those with a high prevalence of health issues and those with low prevalence. This categorization streamlines the process of identifying priority areas for intervention and directing resources effectively. The methodology employed, utilizing R for grouping and ranking, allows for replicability by other researchers who may want to explore additional variables or expand on this framework. Furthermore, the summary of outcomes for policy targeting derived from predictive analytics highlights the potential for implementing various intervention programs, legislative initiatives, and economic development projects aimed at addressing multidimensional socioeconomic and poverty indicators. This approach makes it feasible for policymakers to focus on specific issues faced by different tribes, ensuring that interventions are relevant and impactful. Ultimately, these strategies aim to uplift Indigenous communities, alleviate poverty conditions, and foster economic development, thereby ensuring that these populations are not left behind in broader socio-economic progress. These findings align with existing literature, which argues that addressing these multidimensional deprivations is crucial for fostering sustainable development and improving the welfare of Indigenous populations [6,17,18,56,59,60]. There is an urgent need for targeted policies and programs that address these interconnected issues, focusing on enhancing income opportunities, improving educational access, and ensuring basic health and sanitation services. By doing so, we can work towards breaking the cycle of poverty that has long affected these communities and promote a more equitable future.

## 4 Methods conclusion

This paper has no known restrictions because the data it examines is based on a thorough enumeration of all indigenous houses and populations. It thereby maintains the sample assumptions of internal and external validity and is not subject to sampling error. The multidimensional variables in the data are carefully analyzed and filtered by the writers. Since the multifaceted poverty experienced by indigenous people is so intense and demanding, it is believed to be difficult to quantify. In order to combat poverty and advance economic development, the methods provide useful information for developing policies and initiatives that are especially targeted at the indigenous population. The datasets can be used by other developing countries and underdeveloped regions of the world that are home to indigenous people to gain raw, analyzed, baseline data, and indicators of multidimensional poverty, which can then be used to design strategies for economic development. The data analytics protocols, variables, techniques, policy recommendations, and computational approaches could be employed by future researchers to create similar studies that quantify the intangible components of multidimensional poverty for other Native populations. The poverty analytics methods can be used to compute, validate, and simulate several aspects of poverty, including poverty incidence, poverty gap, severity statistics, watts index, and classifications for indigenous people tribes. The methodology offers indigenous communities comprehensive analytics on various aspects of socioeconomic development. It enables stakeholders like governments, institutions, researchers, and policymakers to develop effective programs for indigenous socio-economic progress globally. Furthermore, the datasets and methods provided can be utilized in future papers to explore the gender context among indigenous people. The current study predominantly used the household as the unit of measurement, analyzing households as collective units comprising male and female members. A paper specifically dedicated to exploring gender-specific poverty and its dimensions could be undertaken. This endeavor could represent a significant step towards understanding indigenous people from a different perspective. Finally, the application of econometric models and predictive analytics datasets may produce empirical data in support of poverty theories pertaining to indigenous populations, adding to the corpus of knowledge regarding the scarcity of multidimensional poverty metrics in the Philippines and other developing countries, particularly for indigenous people.

## CRediT authorship contribution statement

**Emmanuel A. Onsay:** Writing – original draft, Visualization, Validation, Resources, Project administration, Methodology, Investigation, Funding acquisition, Formal analysis, Data curation, Conceptualization. **Jomar F. Rabajante:** Writing – review & editing, Validation, Supervision, Software, Resources, Project administration, Methodology, Investigation, Funding acquisition, Conceptualization.

## Ethics statements

The following ethics statement outlines the ethical considerations and standards observed during the course of this research project.

1. Approval and Consent for Research

The University of the Philippines Los Baños and Partido State University have granted the necessary approvals for the implementation of this study. Additionally, the Local Government Unit of Goa, Camarines Sur has authorized the utilization, analysis, and distribution of processed data results. The conduct of this research is permitted under NTP-2023-S2-C3 of Research Project titled ‘Measuring the Unmeasurable for Poverty Alleviation and Economic Development’.

2. Nature of Data Collection

The human data collected for this study are socio-economic in nature, obtained indirectly from secondary sources, and do not involve any laboratory procedures. Therefore, formal ethical clearances are deemed unnecessary. Furthermore, this investigation does not involve any animal testing, direct participation of human subjects, or data gathered from social media platforms.

3. Declaration of Competing Interests

The authors declare that they have no known competing financial interests or personal relationships that could have influenced the work reported in this paper. This declaration ensures transparency and minimizes any potential biases in the research.

4. Responsible Authorship

All authors listed on this manuscript have significantly contributed to the research and drafting of the report and have agreed to take responsibility for the content. Contributions from non-authors have been acknowledged appropriately.

5. Handling of Confidential Data

Confidentiality has been maintained throughout the research process, ensuring that any sensitive information obtained is used solely with appropriate authorization and consent.

6. Post-Publication Corrections

The authors are committed to maintaining the integrity of the published research. Should any errors be identified post-publication, the authors will address these issues promptly, either by issuing corrections or retracting the article if necessary.

7. Conclusion

These statements reflect our commitment to ethical standards in research and publishing, ensuring integrity, accountability, and transparency in our work.

## Data availability

Onsay, Emmanuel; Rabajante, Jomar, 2023, "Measuring the Unmeasurable Multidimensional Socio-Economic Deprivations and Poverty Predictions: Indigenous People Datasets for Econometrics, Machine Learning, and Quantitative Social Science Modeling", https://doi.org/10.7910/DVN/QSZKUP, Harvard Dataverse, V2.

The dataset provides valuable insights that can inform policy and program development aimed specifically at alleviating poverty and fostering economic growth among indigenous people. Developing countries and poor areas inhabited by indigenous communities can utilize these datasets to gather raw, analyzed baseline data and indicators related to multidimensional poverty, which can help in formulating effective economic development strategies. Future researchers can leverage the analytical protocols, variables, methods, policy recommendations, and computational techniques embedded in the data to conduct comparable investigations into the intangible aspects of multidimensional poverty affecting other Native communities. Metrics such as poverty incidence, poverty gap, severity statistics, the Watts index, and classifications for indigenous tribes can be calculated, validated, and simulated using this poverty analytics dataset. The processed datasets provide descriptive and diagnostic analytics that assess various factors such as population dynamics, health and nutrition, housing and settlement, water and sanitation, basic education from elementary to senior high school, income classifications, employment, livelihood, and community safety for indigenous groups. The prescriptive analytics dataset can aid in program development and impact assessment for the socioeconomic advancement of indigenous populations, both in the Philippines and globally, benefiting local government units, national agencies, indigenous institutions, researchers, scholars, policymakers, academics, philanthropic organizations, and social enterprises. The application of predictive analytics datasets, machine learning algorithms, and econometric models may produce empirical evidence that supports poverty theories related to indigenous populations, enriching the knowledge base concerning the scarcity of multidimensional poverty metrics in the Philippines and other impoverished countries, particularly with respect to indigenous groups [1].

Onsay, Emmanuel; Rabajante, Jomar, 2023, "Measuring the Unmeasurable Multidimensional Socio-Economic Deprivations and Poverty Predictions: Indigenous People Datasets for Econometrics, Machine Learning, and Quantitative Social Science Modeling", https://doi.org/10.7910/DVN/QSZKUP, Harvard Dataverse, V2.

1. DATA DICTIONARY
https://dataverse.harvard.edu/file.xhtml?fileId=7915951&version=1.1
2. INDIGENOUS PEOPLE ANALYTICS https://dataverse.harvard.edu/file.xhtml?fileId=7915947&version=1.1
3. INDIGENOUS PEOPLE TRIBES’ ANALYTICS
https://dataverse.harvard.edu/file.xhtml?fileId=7915944&version=1.1
4. MULTIDIMENSIONAL DATASETS OF INDIGENOUS PEOPLE IN THE 12 TRIBES OF MT. ISAROG, PHILIPPINES
https://dataverse.harvard.edu/file.xhtml?fileId=7915949&version=1.1
5. POPULATION DYNAMICS OF INDIGENOUS PEOPLE
https://dataverse.harvard.edu/file.xhtml?fileId=7915946&version=1.1
6. POVERTY ANALYTICS GRAPH
https://dataverse.harvard.edu/file.xhtml?fileId=7915952&version=1.1
7. POVERTY DATA OF INDIGENOUS PEOPLE
https://dataverse.harvard.edu/file.xhtml?fileId=7915948&version=1.1
8. POVERTY INDICES OF INDIGENOUS PEOPLE
https://dataverse.harvard.edu/file.xhtml?fileId=7915950&version=1.1
9. PRESCRIPTIVE ANALYTICS
https://dataverse.harvard.edu/file.xhtml?fileId=7915945&version=1.1
10. COMPREHENSIVE MULTIDIMENSIONAL DATASETS OF IP
https://dataverse.harvard.edu/file.xhtml?fileId=10267173&version=2.0
11. COMPREHENSIVE POVERTY DATA OF IP

https://dataverse.harvard.edu/file.xhtml?fileId=10267174&version=2.0.

A part of this work had to be published as a prize in the proceedings of an Asian international research competition (IRC), where the paper won grand prize. However, the paper only addresses health dynamics. This work and its methods are detailed and comprehensive; they include various interacting variables that have not yet been fully explored as well as multidimensional constructs and indicators. The techniques used in this work combine advanced econometrics and machine learning, making them highly valuable for future research:

Onsay, E. A. (2022). Poverty profile and health dynamics of indigenous people. Int Rev Soc Sci Res, 2, 1–27. Proceedings of the IIARI International Research Competition - Grand Winner Researcher 2021. https://iiari.org/onsay-leads-the-iiari-research-competitions-2021/

Discussion on the simplified applications of these methods can be accessed thoroughly in another Elsevier journal publication:

Onsay, E. A., & Rabajante, J. F. (2024). When machine learning meets econometrics: Can it build a better measure to predict multidimensional poverty and examine unmeasurable economic conditions? Science Talks, 11, 100387. https://doi.org/10.1016/j.sctalk.2024.100387.

## Funding

This work is funded by the Department of Agriculture (DA) and 10.13039/501100007421University of the Philippines Los Baños (UPLB) Accelerating Growth to One Research and Extension in Action (AGORA) and Agricultural and 10.13039/100020664Rural Development Scholarship (ARDS)

## Declaration of competing interest

The authors declare that they have no known competing financial interests or personal relationships that could have appeared to influence the work reported in this paper.
